# PHI-Nets: A Network Resource for Ascomycete Fungal Pathogens to Annotate and Identify Putative Virulence Interacting Proteins and siRNA Targets

**DOI:** 10.3389/fmicb.2019.02721

**Published:** 2019-12-06

**Authors:** Elzbieta I. Janowska-Sejda, Artem Lysenko, Martin Urban, Chris Rawlings, Sophia Tsoka, Kim E. Hammond-Kosack

**Affiliations:** ^1^Department of Biointeractions and Crop Protection, Rothamsted Research, Harpenden, United Kingdom; ^2^Department of Computational and Analytical Sciences, Rothamsted Research, Harpenden, United Kingdom; ^3^Department of Informatics, Faculty of Natural and Mathematical Sciences, King’s College London, London, United Kingdom

**Keywords:** biological networks, pathogenic fungi, interactome inference, small interfering RNA, PHI-base, gene function inference

## Abstract

Interactions between proteins underlie all aspects of complex biological mechanisms. Therefore, methodologies based on complex network analyses can facilitate identification of promising candidate genes involved in phenotypes of interest and put this information into appropriate contexts. To facilitate discovery and gain additional insights into globally important pathogenic fungi, we have reconstructed computationally inferred interactomes using an interolog and domain-based approach for 15 diverse Ascomycete fungal species, across nine orders, specifically *Aspergillus fumigatus*, *Bipolaris sorokiniana*, *Blumeria graminis* f. sp. *hordei*, *Botrytis cinerea*, *Colletotrichum gloeosporioides*, *Colletotrichum graminicola*, *Fusarium graminearum*, *Fusarium oxysporum* f. sp. *lycopersici*, *Fusarium verticillioides*, *Leptosphaeria maculans*, *Magnaporthe oryzae*, *Saccharomyces cerevisiae*, *Sclerotinia sclerotiorum*, *Verticillium dahliae*, and *Zymoseptoria tritici*. Network cartography analysis was associated with functional patterns of annotated genes linked to the disease-causing ability of each pathogen. In addition, for the best annotated organism, namely *F. graminearum*, the distribution of annotated genes with respect to network structure was profiled using a random walk with restart algorithm, which suggested possible co-location of virulence-related genes in the protein–protein interaction network. In a second ‘use case’ study involving two networks, namely *B. cinerea* and *F. graminearum*, previously identified small silencing plant RNAs were mapped to their targets. The *F. graminearum* phenotypic network analysis implicates eight *B. cinerea* targets and 35 *F. graminearum* predicted interacting proteins as prime candidate virulence genes for further testing. All 15 networks have been made accessible for download at www.phi-base.org providing a rich resource for major crop plant pathogens.

## Introduction

Global food security is threatened by numerous plant disease-causing fungal pathogens, which infect agricultural and horticultural crops. New control mechanisms are urgently needed as pathogens (i) evolve resistance to the ever-narrowing range of available site specific and broad-spectrum fungicides, and (ii) regularly overcome the various disease resistance genes introduced by plant breeders. Due to their economic and societal importance, plant pathogens are intensively studied using molecular biology and molecular genetic research tools and approaches. In addition, over the past 15 years, whole genome information has become available for the most problematic plant pathogenic species and more recently such datasets have been augmented with genomes from additional individual strains possessing a range of different biological properties. The ‘Top 10’ fungal pathogens identified based on their scientific and economic importance include fungi with a wide diversity of lifestyles (Dean et al., 2012). For example, the necrotrophic *Botrytis cinerea* kills infected plant cells outright, whereas hemibiotrophic fungi such as *Magnaporthe oryzae*, *Fusarium graminearum*, *Fusarium oxysporum*, *Colletotrichum* spp., and *Zymoseptoria tritici* invade initially living host tissue until host cell death occurs. Biotrophic fungi, such as *Blumeria graminis*, keep host plants alive throughout the disease formation process. In addition, some pathogens (*Colletotrichum* spp.) can either infect a wide range of crop species or are specialists that infect just a single crop species (*B. graminis* f. sp. *hordei*). Differences in gene content of filamentous fungal pathogens can be attributed to the action of repetitive elements, transposons, and genome rearrangements in several lineages (Raffaele and Kamoun, 2012).

Development of effective and resilient control strategies for infectious diseases caused by pathogenic fungi relies on an in-depth understanding of the underlying biological processes (BPs) and knowledge of potential points where these processes can be disrupted. This type of data is commonly collected experimentally using targeted gene modification and/or gene-silencing experiments, where observed phenotypes relate specifically to changes in key points during virulence and pathogenicity. One of the resources curating phenotypic disease outcomes of gene modification experiments with a particular emphasis on plant pathogenic fungi of agricultural and horticultural significance is the Pathogen–Host Interactions database (PHI-base^1^) (Urban et al., 2017). Importantly, PHI-base collects data from both positive- and negative-experimental outcomes. However, to understand the underlying mechanisms of observed phenotypes, and to identify proteins contributing to virulence it is important to consider them in the context of networks of molecular interactions, where proteins of unknown function can be targeted. Even in the well-studied, non-pathogenic filamentous fungal model species *Neurospora crassa*, only ∼60% of proteins are annotated (Ellison et al., 2014). Therefore, scope exists for knowledge transfer from model species to less studied species, where extensive molecular interaction information is available (such as the yeasts *Saccharomyces cerevisiae* and *Schizosaccharomyces pombe*, the worm *C. elegans*, fruit-fly *D. melanogaster*, and the mouse *M. musculus*).

The potential to use protein–protein interaction network analysis to decipher pathogenicity and virulence mechanisms as well as identify candidate genes has been a topic of active research during the last decade (reviewed in Cairns et al., 2016). In these applications, a biological network is usually constructed by linking together biological entities that either interact physically (e.g., protein–protein interaction, enzyme binding a substrate) or are shown to be associated with a more abstract experimentally derived common property (e.g., co-expression or co-localization). When insufficient experimental data is available to construct a network, inference from other related data types may be used instead. Two common computational methods to infer protein–protein interaction (PPI) networks are (i) the interolog approach relying on sequence similarity between proteins from different species and (ii) the domain-based approach with a focus on conserved Pfam domains (Li and Zhang, 2016).

The approaches for identifying promising candidates in pathogenic fungi using biological networks so far have primarily focused on exploiting the ‘guilt-by-association’ principle, most often by employing either a ‘direct neighborhood’ or a community structure detection strategy. The direct neighborhood approach considers a set of nodes directly connected to each potential target and prioritization is based on a score related to the number of known annotations among them. This score may be further adjusted by applying a weight to incorporate additional factors like confidence in links or expression patterns. In a community structure detection approach the network is partitioned into distinct communities, modules or clusters according to its pairwise links that define the network topological structure. Then, distribution of annotated nodes in those modules is explored further by methods of enrichment analysis and prioritization of genes is based on module membership and overall score of the module.

For filamentous fungi, predicted protein–protein interactions were previously explored for several non-pathogenic and pathogenic species. Networks exist for *Neurospora crassa* (Wang et al., 2011) and human-infecting fungi *Candida albicans*, *Aspergillus fumigatus*, and *Cryptococcus neoformans* (Kim H. et al., 2015; Remmele et al., 2015). Additional networks are available for a few plant pathogenic species including *Magnaporthe grisea* (He et al., 2008), *Phomopsis longicolla* (Li et al., 2018), *Rhizoctonia solani* (Lei et al., 2014), *Fusarium verticillioides* (Kim M. et al., 2015), and *F. graminearum* (Zhao et al., 2009; Liu et al., 2010; Bennett et al., 2012; Lysenko et al., 2013). However, the approaches used differed across studies and do not allow comparative network investigation. In addition, early genome assemblies were used, i.e., *F. graminearum*, that now require rebuilding of the underlying interactomes.

Studies during the last decade on plant–pathogen interactions identified a novel host defense-mechanism in animals and plants, called cross-kingdom/organism RNA interference (RNAi) (Weiberg et al., 2013; Weiberg and Jin, 2015; Cai et al., 2018). Mobile small silencing RNAs (siRNAs) produced by the hosts are transferred to the pathogen during the invasion process and attenuate virulence. For the *Arabidopsis*-*B. cinerea* pathosystem, 42 *Arabidopsis* siRNAs were detected in *B. cinerea* protoplasts generated from infected *Arabidopsis* plants. These siRNAs implicated 21 putative targets in *B. cinerea* targeting several global BPs including vesicle transport, transcription and signal transduction. However, most of the putative targets have no associated phenotype, and their function and potential protein interaction partners are unknown due to the lack of published functional gene tests in *B. cinerea*. In contrast, for *F. graminearum* which causes disease on many cereal species, a wealth of phenotype information exists. Here initial studies suggest that wheat plants also utilize host RNAi suppression of genes within the attacking pathogen (Chen et al., 2016; Jiao and Peng, 2018).

To further advance mechanistic understanding of fungal virulence and pathogenicity for plants, increasingly comparative analyses are performed using selected groups of pathogenic species with similar or contrasting lifestyle strategies or host ranges. For network-based analyses to become an effective part of these comparative studies, the availability of networks for multiple species built in the same way is urgently required. Similarly, since the recent identification of two-way cross-kingdom siRNA trafficking as a potential new route for communication and manipulation in host–fungal interactions, the sequences targeted by siRNA also need to be formally recognized and displayed within these networks.

The main aims of this study were therefore three-fold. Firstly, we built a series of protein domain–domain networks for pathogenic ascomycete fungi of global importance to agriculture and horticulture. Within each network, all phenotypic and ontology information for the 10s to 1000 + nodes formally tested for a role in virulence would be placed. Free access to this suite of network datasets would permit specialists and non-specialists alike to develop a multitude of interdisciplinary approaches to investigate virulence and pathogenicity processes in a network context. Second, we elucidated the relationship between the well-studied proteins and metabolites linked to virulence and pathogenicity, and the newly emerging field of small interfering RNAs modulating the outcome of host–pathogen interactions. Third, we used two exemplar species, a highly studied pathogen and a less-studied pathogen, to illustrate how such network resources can facilitate the identification of key interactions and possible candidate virulence and pathogenicity genes with hitherto minimal to no formal annotation.

## Materials and Methods

### Construction of Predicted Protein–Protein Interaction Networks

**FIGURE 1:**
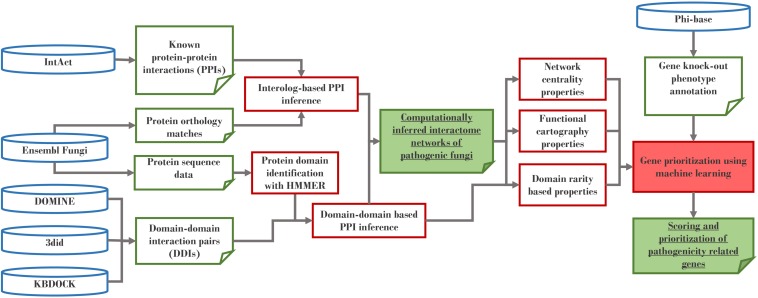
Construction of computationally-inferred interactomes.

The predicted interactomes were constructed using an interolog and domain–domain interaction (DDI) approach (Figure 1). The interolog approach works under the assumption that if a pair of proteins in one species are experimentally confirmed to interact, this protein–protein interaction is also likely to be conserved for their orthologs in another species. Therefore, this method requires reference interactome(s) and orthologous sequences mappings that could link them to a species of interest. We have chosen non-pathogenic Ascomycetes *S. cerevisiae* and *S. pombe* as two reference interactome species, because both species have some of the best-profiled, experimentally verified interactomes. Our data for these two species was taken from the EBI IntAct database (May 2016 release) (Orchard et al., 2014) and was combined with orthologs retrieved from Ensembl Fungi (May 2016 release) (Kersey et al., 2016), which were originally derived using Ensembl Compara pipeline (Herrero et al., 2016).

The DDI approach operates under the premise that some of the interactions are mediated by specific protein domains and can therefore be assumed to also occur between proteins that possess these domain pairs. Several public databases identify such interacting domain pairs using protein 3D structure analysis and statistical approaches. To obtain the most complete set we have integrated the data from three DDI databases: KBDOCK (Ghoorah et al., 2014), DOMINE (Yellaboina et al., 2011), and 3did (Stein et al., 2005). Computational scripts were made available at https://github.com/PHI-base/phi-nets/.

Complete genomes for the 15 fungi explored in this study were obtained from Ensembl Fungi version 31^2^ (Supplementary Table S1). The domain repertoire for each species proteome was identified using the HMMER algorithm which is based on biosequence analysis using profile hidden Markov models (Eddy, 2009), implemented on TimeLogic^®^ HMM (Hidden Markov Models) version 8.7 and domain models from Pfam database (version 29.0) (Finn et al., 2016). For each of the 15 proteomes, additional processing of the raw HMMER output was performed using a custom python script to resolve overlapping domain issues. The general rule for solving the domain overlapping problem was adopted from previous work (Seidl et al., 2011) as follows: for non-overlapping domains in the given protein the score of −1 was assigned and the domain remained in the protein. In complex situations where multiple domains overlapped, the set of overlapping domains was represented as an adjacency matrix, where the scores were assigned as per application of the rules. Specifically, a score of 1 was assigned to the row of predicted domain if the rules pointed toward this domain as better, compared to the domain in the column, and a 0 if the situation was the other way around. The domain with the score equal to 1 remained in the protein, whereas the domain with the score equal to 0 was removed from the protein sequence. Although, this approach resolved the overlap in most cases, there were proteins where the overlaps had to be resolved manually (Supplementary Information S1). This non-redundant dataset was then used to infer interactions for each pair of proteins containing interacting domains included in at least one of the three DDI databases.

### Quality Evaluation of Predicted Interactomes

To verify the quality of predicted interactions we have calculated summary statistics for the number of predicted interacting partners found in the same cellular compartment and functional similarity according to the Gene Ontology (GO) annotation in biological process (GO-BP) and molecular function (GO-MF) (Ashburner et al., 2000) (release May 2016). In all these cases we have used gene-level annotation from the Ensembl Fungi BioMarts (Kinsella et al., 2011). These annotations were compared to two reference sets: random control where the same number of random annotated gene pairs were created for each of the 15 species, and an experimentally verified set of interactions for *S. cerevisiae*. The estimated correctness of inferred interactions was evaluated using two metrics: major cellular compartment co-localization and similarity of BP annotations. For the former, a pair of proteins was considered co-localized if both predicted proteins were annotated with one of the following eight major compartment terms (or its subclass descendants): ‘extracellular region,’ ‘cytoplasm,’ ‘nucleus,’ ‘mitochondrion,’ ‘endoplasmic reticulum,’ ‘Golgi apparatus,’ ‘fungal-type vacuole,’ and ‘fungal-type cell wall’. For the latter, the similarity of GO annotations was measured using a semantic similarity approach, which uses mutual information content of the most informative common ancestor GO annotation term (Lord et al., 2003).

### Integration of PHI-Base Annotation

Pathogen–host interactions database is a unique resource that focuses on genes involved in pathogen–host interactions, and gene functions that are experimentally verified. Annotations are supported by strong experimental evidence (gene disruption, gene silencing, or other alteration experiments). PHI-base version 4.6 (November 2018 release) was used to annotate the predicted proteins in the 15 Ascomycete networks. In general, nine high level phenotyping terms are used to describe the phenotype outcome for one interaction in PHI-base: loss of pathogenicity, reduced virulence, unaffected pathogenicity, increased virulence, effector gene (plant avirulence determinant), lethal, enhanced antagonism, resistant to chemical, sensitive to chemical (Urban et al., 2015). In our analysis we summarized these terms in three groups of phenotyping terms, namely ‘pathogenicity-related,’ ‘pathogenicity-unrelated,’ and ‘mixed outcome.’ The ‘pathogenicity-related’ annotation consists of ‘loss of pathogenicity,’ ‘reduced virulence,’ and ‘increased virulence’ phenotyping terms, whereas ‘unaffected pathogenicity’ phenotype represents a pathogenicity-unrelated set. In PHI-base one or more interactions with a host species can be assigned to a given gene. This creates situations where a gene is linked to several contrasting phenotypic outcomes. In this study we classified such phenotype as ‘mixed outcome.’ Other PHI-base phenotyping terms were not useful in our analysis. The term ‘lethal’ is not supported with experimental evidence in PHI-base.

### Topological Proximity to Proteins With Characterized Phenotypes

We have used a random walk with restart (RWR) (Köhler et al., 2008) method to identify likely candidate genes within the ‘pathogenicity related’ group. Random walk with restart calculates the probability of a node in the network being visited by a random walker which starts with equal probability from any of the nodes in a seed set. At each step the walker also has a defined probability of restarting the walk from one of the seed nodes. This method has been demonstrated to be very successful for prioritization of disease-associated genes in human protein–protein interaction networks. However, to the best of our knowledge this is the first time it has been used to predict a pathogenicity phenotype in pathogenic fungi. The advantage of this method is that it can be used to produce a score for protein nodes without direct connections to proteins with characterized phenotypes. The method also considers a wider neighborhood of a node, like overall distribution of nodes in the neighborhood, as well as degrees and edge densities of the surrounding nodes. For this study we have calculated an exact solution, e.g., the set of probabilities to which it will converge to after an infinite number of iterations, calculated according to the formula from (Smedley et al., 2014). In each case, two sets of RWR scores were computed, using either genes in the known pathogenicity-related/unrelated categories as the seeds. The inference potential of these results was evaluated using standard area under the receiver-operator curve (ROC-AUC). Briefly, the ROC-AUC analysis is used in machine learning to evaluate the performance of a binary classifier, its ability to correctly order ‘true’ and ‘false’ results with some score (e.g., a probability returned by classifier for an instance to be of ‘true’ class). The ROC-AUC value of 0.5 would indicate that the prediction quality is the same as random chance, whereas 1.0 would mean a perfect prediction.

### Modularity and Functional Cartography Analysis

The modular structure of all networks was profiled using the Louvain graph clustering algorithm (Blondel et al., 2008). As biological networks are known to be organized into communities that may also exhibit hierarchical structure, cluster assignments at different levels of granularity are potentially informative. To explore and optimize cluster granularity, we have applied the Louvain algorithm recursively to further break down larger clusters above a certain size threshold and which are not fully connected cliques. To optimize this threshold, we have performed a scan across a 5–200 size range and examined the trade-off between purity (defined as proportion of nodes with the same annotation with respect to virulence) and the Shannon entropy of the resulting modules (relative to splitting of each virulence annotation category into smaller subsets) with respect to pathogenicity-related genes of the 15 species. According to this analysis, the size of 50 was found to be at the best trade-off point between these two metrics.

The functional cartography analysis characterizes nodes according to their roles in a given community (Guimera and Nunes Amaral, 2005). Here, the analysis was performed for the largest connected component of each network. Prior to the cartography analysis, the Louvain clustering algorithm was used to detect communities within the largest connected component of the given network. The cartography analysis primarily considered the following two properties: within-module connectivity (z-normalized within module degree) and participation coefficient (proportion of links a node has to members of other modules). Based on the region in a parameter space of z-score and participation coefficient, nodes were categorized as hubs and non-hubs and the seven following categories were identified within each of the networks in this study: R1 – ultra-peripheral node, R2 – peripheral node, R3 – non-hub connector node, R4 – non-hub kinless node, R5 – provincial hub, R6 – connector hub and R7 – global kinless hub (Supplementary Information S2). The role of the nodes was determined using GIANT version 1.0 plugin for Cytoscape version 3.7.1. Following the identification of the nodes’ role within the first connected component of each network, the association of the node role (position) with fungi lifestyle was tested with the aid of a chi-square test.

### Analysis of *B. cinerea* RNA Silencing Targets in *F. graminearum* and *B. cinerea* Networks Using Cytoscape

Web-based BLAST provided by Ensembl Fungi^3^ was used to map the 33 siRNA target genes identified in *B. cinerea* strain B05.10 (Cai et al., 2018) to the latest *B. cinerea* genome assembly GCA_00143535.4. Orthologs between *B. cinerea* and *F. graminearum* strain PH-1 were identified using BioMart (Kersey et al., 2018). *B. cinerea* and *F. graminearum* networks were additionally annotated using phenotypes provided by PHI-base release version 4.6. For *F. graminearum*, gene names for the subnetworks were taken from FusariumMutantDb (Baldwin et al., 2018). Complexity in *B. cinerea* and *F. graminearum* networks was reduced by dividing them first into Louvain modules. Next, genes of interest (*B. cinerea* targets/orthologs and genes with PHI-base annotation) and their first-neighbors were selected using list-selection in Cytoscape.

## Results

### Inferred Interactomes of Pathogenic Fungi

In total 15 globally important Ascomycete fungal species across nine taxonomic orders were selected for network analysis. Of these, 13 are serious plant pathogenic species with different *in planta* lifestyles and host ranges, one is a serious human pathogen with a prominent saprophytic phase in multiple environments and the last is the model species *S. cerevisiae* (Table 1). For each species the percentage of proteins in the predicted proteomes with one or multiple domains was predicted (Table 2). The protein–protein interactions were inferred using DDI and interolog approaches. The sets of DDIs were taken from KBDOCK, DOMINE, and 3did interacting domains databases. The interologs where inferred by taking experimentally established interacting orthologous protein pairs in *S. cerevisiae* and *S. pombe* and combining them with experimental interaction data from the IntAct database (Orchard et al., 2014). The overall number of edges inferred from each of these resources is shown in Table 3. Across all 15 species explored, the DDI-inferred interactions had the highest overall coverage (from ∼70 to 100%), with contributions from KBDOCK and 3did being particularly prominent (Table 4). The coverage by the interolog-inferred interactions was considerably lower within the range 7.92–32.59% of all predicted interactions.

**TABLE 1:** Lifestyle, host range, and PHI-base network annotations for the 15 selected fungal species.

**Order**	**Species**	**NCBI taxonomy identifier**	**Lifestyle**	**Host species types (natural)**	**No of plant hosts; Vast – well over 100 host species, Many – up to 100 host species, A few – up to 20 host species, One – a single host species**	**No of different host interactions recorded in the literature^3, 4^**	**PHI-base annotations in network**
*Eurotiales*	*Aspergillus fumigatus*	746128	Lung infections and invasive aspergillosis (IA)^1^	Human, domesticated and wild animal and bird species^1^	Many	Footnote ^2^	114
*Pleosporales*	*Bipolaris sorokiniana*	45130	Hemibiotroph	Cereal Monocot	Vast	374	2
*Erysiphales*	*Blumeria graminis* f. sp. *hordei*	62688	Obligate biotroph	Cereal Monocot	One	1	1
*Helotiales*	*Botrytis cinerea*	40559	Hemibiotroph – necrotroph	Cereal Monocot – Non-Cereal Monocot – Dicot	Vast	1367	50
*Glomerellales*	*Colletotrichum fructicola ^6^*	690256	Hemibiotroph – necrotroph	Non-Cereal Monocot – Dicot	Vast	1911 ^5^	2
*Glomerellales*	*Colletotrichum graminicola*	31870	Hemibiotroph	Cereal Monocot and Dicot	Vast	342	8
*Hypocreales*	*Fusarium graminearum*	5518	Hemibiotroph – necrotroph	Cereal Monocot – Non-Cereal Monocot – Dicot	Vast	216	789
*Hypocreales*	*Fusarium oxysporum* f. sp. *lycopersici*	59765	Necrotroph	Dicot	A few	15	26
*Hypocreales*	*Fusarium verticillioides*	117187	Hemibiotroph – necrotroph	Cereal Monocot – Non-Cereal Monocot – Dicot	Many	124	24
*Pleosporales*	*Leptosphaeria maculans*	5022	Hemibiotroph – necrotroph	Dicot	Vast	110	2
*Magnaporthales*	*Magnaporthe oryzae*	318829	Hemibiotroph	Cereal Monocot	Many	46	389
*Saccharomycetales*	*Saccharomyces cerevisiae*	4932	Saprotroph	None	Zero	0	13
*Helotiales*	*Sclerotinia sclerotiorum*	5180	Necrotroph	Non-Cereal Monocot – Dicot	Vast	684	3
*Glomerellales*	*Verticillium dahliae*	27337	Necrotroph	Dicot	Vast	395	25
*Capnodiales*	*Zymoseptoria tritici*	1047171	Hemibiotroph	Cereal Monocot	A few	33	13

*^1^IA disease only in human and animal hosts with severe immunodeficiency; ^2^*Aspergillus* and aspergilloses in wild and domestic animals: a global health concern with parallels to human disease (Seyedmousavi et al., 2015); ^3^https://nt.ars-grin.gov/fungaldatabases/fungushost/fungushost.cfm; ^4^http://www.plantwise.org/KnowledgeBank; ^5^Host species noted for *Colletotrichum gloeosporioides* in database 3, ^6^*Colletotrichum fructicola* previously known as *Colletotrichum gloeosporioides*.*

**TABLE 2:** Summary of protein domain annotation statistics for the genome versions used in this study.

**Species**	**Genome version^1^**	**Predicted proteins count**	**Count of proteins with a domain**	**% exome with a domain**	**% exome with multiple domain**	**% exome in the DDI network^2^**
						
*Aspergillus fumigatus*	CADRE.31	9630	6989	72.58	21.50	52.56 (33.33/19.23)
*Bipolaris sorokiniana*	nd90pr.Cocsa1.31	12214	7416	60.72	17.70	44.12 (28.20/15.92)
*Blumeria graminis* f. sp. *hordei*	EF1.31	6470	4337	67.03	21.42	46.24 (27.73/18.52)
*Botrytis cinerea*	ASM15095v2.31	12103	7691	63.55	18.49	46.00 (29.57/16.43)
*Colletotrichum fructicola*^3^	GCA_000319635.1.31	15381	9838	63.96	16.60	46.93 (31.86/15.07)
*Colletotrichum graminicola*	GCA_000149035.1.31	12020	7816	65.02	18.59	46.97 (30.27/16.71)
*Fusarium graminearum*	RR.26	14164	8488	59.93	17.22	43.79 (28.30/15.49)
*Fusarium oxysporum* f. sp. *lycopersici*	FO2.31	17696	9805	55.41	14.08	41.10 (28.55/12.55)
*Fusarium verticillioides*	ASM14955v1.31	14185	8286	58.41	15.54	43.26 (29.18/14.08)
*Leptosphaeria maculans*	ASM23037v1.31	12469	6234	50.00	15.16	35.94 (22.51/13.43)
*Magnaporthe oryzae*	MG8.31	12755	7242	56.78	16.47	40.98 (26.21/14.77)
*Saccharomyces cerevisiae*	R64-1-1.31	6705	4837	72.14	23.15	50.16 (30.08/20.07)
*Sclerotinia sclerotiorum*	ASM14694v1.31	10175	4568	44.89	13.53	30.50 (19.27/11.22)
*Verticillium dahliae*	GCA_000150675.1.31	10535	6867	65.18	18.35	46.39 (30.19/16.20)
*Zymoseptoria tritici*	MG2.31	10931	6597	60.35	17.23	43.77 (28.64/15.12)

*^1^All genomes were obtained from Ensembl Fungi v.31; ^2^The percentages in brackets refer to single/multiple domain sub-counts respectively; ^3^*Colletotrichum fructicola* previously known as *Colletotrichum gloeosporioides*.*

**TABLE 3:** Network statistics.

**Species**	**Nodes**	**Edges**	**Average clustering coefficient**	**Average degree centrality**	**Modularity of the network**	**Number of CCs**	**Nodes in the largest CC**	**Edges in the largest CC**	**Communities in the largest CC (Louvain)**	**Modularity of the largest CC**
*Aspergillus fumigatus*	5925	277441	0.631	93	0.4998	117	5498	276432	34	0.4974
*Bipolaris sorokiniana*	5389	264403	0.784	98	0.5117	258	4302	260418	32	0.5093
*Blumeria graminis* f. sp. *hordei*	3816	154218	0.477	80	0.3571	35	3709	153965	16	0.3363
*Botrytis cinerea*	6416	344586	0.651	107	0.5087	130	5910	342596	30	0.5064
*Colletotrichum fructicola*^1^	8161	444775	0.699	109	0.6430	137	7343	439356	47	0.6321
*Colletotrichum graminicola*	6514	297282	0.649	91	0.5482	128	5946	294921	38	0.5442
*Fusarium graminearum*	7062	381518	0.663	108	0.5748	130	6494	379470	38	0.5689
*Fusarium oxysporum* f. sp. *lycopersici*	8292	452631	0.699	85	0.6224	146	7571	449448	43	0.6177
*Fusarium verticillioides*	7094	334015	0.675	94	0.5636	141	6472	331647	42	0.5707
*Leptosphaeria maculans*	5327	221687	0.600	83	0.4423	97	4951	220656	27	0.4388
*Magnaporthe oryzae*	6071	287159	0.632	94	0.5065	119	5574	285379	32	0.5021
*Saccharomyces cerevisiae*	6024	235631	0.389	78	0.3502	3	6020	235629	11	0.3420
*Sclerotinia sclerotiorum*	3803	118987	0.616	62	0.4486	86	3531	118393	26	0.4351
*Verticillium dahliae*	5801	247581	0.637	85	0.4968	113	5282	245569	34	0.4763
*Zymoseptoria tritici*	5609	251215	0.621	88	0.4495	104	5202	250084	31	0.4485

*CC, connected component; CCs, connected components; ^1^*Colletotrichum fructicola* previously known as *Colletotrichum gloeosporioides*.*

**TABLE 4:** Summary of edges generated from each of the data sources across all 15 predicted interactome networks.

**Inferred interaction source**	**Number of edges**	**Min/max proportion in individual networks**
DOMINE	2,652,834	58.56–73.88%
3did	2,072,939	31.38–65.21%
KBDOCK	755,866	10.11–30.10%
Overall (DDI):	3,579,922	69.68–100.00%
From *S. cerevisiae*	542,595	0.0–32.45%
From *S. pombe*	9,086	0.0–0.65%
Overall (interolog):	548,750	7.92–32.59%

*For combined counts and proportions, the numbers were done on non-redundant edge sets of those super-types.*

There was considerable variation in the sizes of the reconstructed networks (Table 3, Raw data in Supplementary Table S2). The largest reconstructed network was for *F. oxysporum* f. sp. *lycopersici* (8,292 nodes and 45,2631 edges), which reflects the far larger number of genes predicted for this species as well as the 2nd largest number of proteins with at least one domain predicted (Table 2). At the other extreme the two smallest reconstructed networks were for *Sclerotinia sclerotiorum* (3,803 nodes and 118,987 edges) and *B. graminis* f. sp. *hordei* (3,816 nodes and 154,218 edges). *S. sclerotiorum* had the lowest percentage of the exome with a predicted domain (∼45%), whereas the obligate biotroph *B. graminis* f. sp. *hordei* is known to have a very restricted exome compared to numerous non-biotrophic plant pathogenic species (Spanu et al., 2010). The remaining species corresponded to networks of a broadly similar size. The brassica-infecting *L. maculans* and *S. sclerotiorum* had a low percentage of the exome with a predicted domain in the reconstructed network (Table 2), as well as a low number of proteins with at least one domain predicted.

To explore the locations of the PHI-base genes in each of the networks, the total gene list downloaded from PHI-base 4.6 with the original curator annotation was partitioned into three logical categories, namely (a) pathogenicity/virulence required, termed ‘pathogenicity – related’ (b) pathogenicity/virulence not required, termed ‘pathogenicity-unrelated’ and (c) pathogenicity context dependent, i.e., only required for the infection of certain plant host species and/or tissue types, termed ‘mixed outcome.’ As expected, the number of PHI-base annotated proteins found in each of the 15 reconstructed networks was generally proportional to the number of original annotations available for that species (Table 1). In total, of the 1,461 PHI-base annotated genes with phenotypes, 1,362 (93%) were included in one or more of the 15 inferred interactome networks, of which 569 were required for pathogenicity/virulence, 726 were not required for pathogenicity/virulence and 67 had a pathogenicity context specific phenotype. For 6 species (*A. fumigatus, B. cinerea, F. graminearum, F. oxysporum, F. verticillioides*, and *M. oryzae*) context-specific pathogenicity nodes were present within the network. For the other networks, only a single type of bioassay had been used by the international community, for example only a wheat leaf bioassay is used to explore *Z. tritici* virulence requirements, or that the gene sequence involved lacked either a domain or a domain interaction. The four most populated inferred interactome networks, in decreasing order of abundance, were *F. graminearum*, *M. oryzae, A. fumigatus*, and *B. cinerea*. These four species have the highest PHI-base annotation of the 15 species selected, again in decreasing order of abundance.

### Quality Evaluation of Predicted Interactomes

**FIGURE 2:**
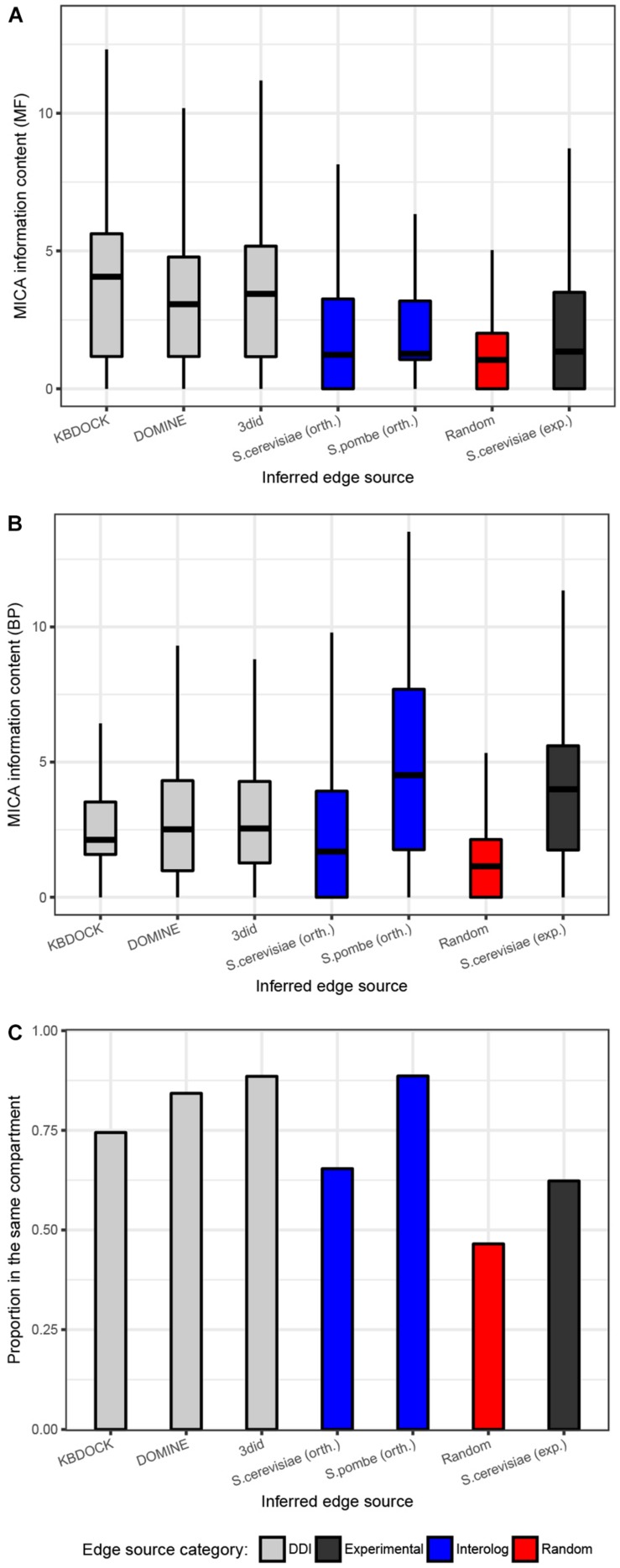
Quality evaluation of the 15 predicted protein–protein interaction networks for pathogenic fungi. **(A)** Functional similarity was quantified using the information content for the most informative common ancestor Gene Ontology term for the linked proteins in the biological process (BP). **(B)** Molecular function (MF) aspects of the gene ontology. **(A,B)** Shows the (overall functional similarity for interacting pairs. Data are presented using a Tukey style box-an- whisker plot indicating the median as a horizontal line. **(C)** Proportions of all interaction pairs co-localized to the same compartment. Edge evidence sources are indicated by colors: Gray = inferred from domain pairs known to interact, black = experimentally-determined, blue = inferred from interacting ortholog pairs, red = baseline made up from randomly picked pairs of proteins of the same species.)

To evaluate the quality of the different sources of inferred interactions, we have explored the numbers of co-localized interaction partners and the semantic similarity of their functional annotations in BP and molecular function (MF) aspects of the Gene Ontology (GO). This analysis was performed on all the 15 reconstructed networks and used respective GO annotation for each of the species from Ensembl Fungi database (Kersey et al., 2018). The expected pattern is that true positive interactors would be found in the same compartment and be functionally similar. The distributions of edges from each source were compared to the set of randomly drawn pairs and experimentally confirmed interactions from *S. cerevisiae* (Figure 2). As expected, the random control had on average substantially lower semantic similarity and the lowest proportion of co-localized interaction partners. The subsets generated from the three DDI resources were quite similar in terms of semantic similarity for both BP and MF aspects. Interestingly, these subsets had a much higher proportion of co-localized interactors and MF similarity compared to experimental interactions from *S. cerevisiae*. This is likely due to the substantial number of high-throughput interaction studies included in the latter experimental data set, which may yield substantial numbers of false-positive interactions. The *S. cerevisiae* orthology-inferred subset of interactions appears to follow the same pattern as the experimental one, though *S. pombe*-inferred subsets appear to score much higher with respect to both co-localization and BP semantic similarity. The quality of interaction networks can therefore be validated by comparing an average functional similarity score of predicted links to an average of a randomly drawn set of a similar size.

### Random Walk With Restart Analysis

**FIGURE 3:**
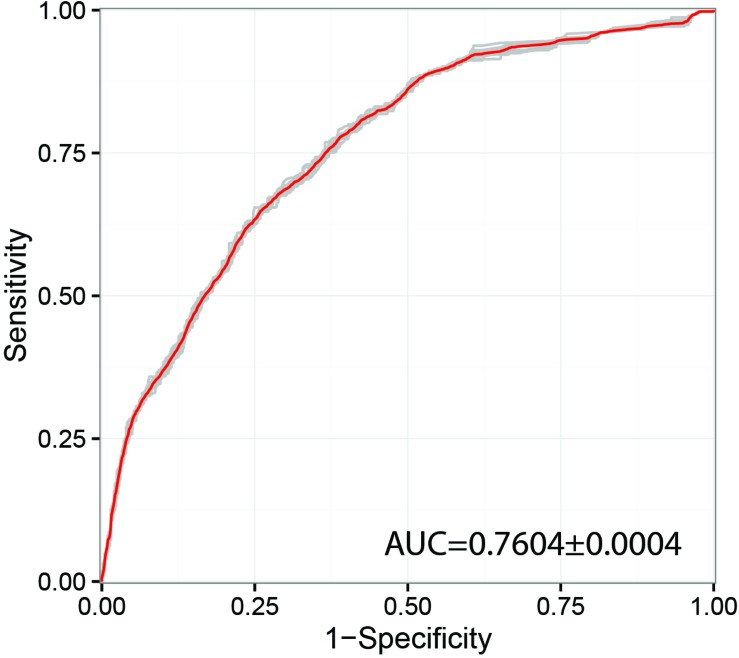
Receiver operating characteristic curve (ROC) used for Random walk with restart (RWR) from known pathogenicity-related and pathogenicity-unrelated seeds combined using random forest algorithm. The model was trained on the dataset of the four most well-annotated species and evaluated using 5-fold cross validation. AUC – area under curve.

Previous studies have shown that network propagation approaches can be highly promising for prioritization of human disease (genetic disorder) genes (Köhler et al., 2008) and profiling of cancer mutation patterns (Leiserson et al., 2015). However, until now applications of these methods were focused in biomedical domains and potential applications for pathogenic species of agricultural interest has not been widely explored. In this study we have investigated the performance of the random walk with restart (RWR) algorithm for prioritization of genes likely to produce a pathogenicity-related phenotype in gene deletion or gene silencing experiments. Only the most populated inferred interactome network with a total of 676 PHI-base gene entries was selected for this type of analysis, namely *F. graminearum*. With regards to the predictive power of the method, the receiver-operator curve (ROC) showed an area under the curve (AUC) of 0.76 (Figure 3), which indicates acceptable prediction. This metric can be compared to other similar RWR studies, for example in the human disease gene prediction study (Köhler et al., 2008) a ROC-AUC score of 0.981 was obtained using the RWR method, whilst the cancer mutation study successfully identified significant clusters of somatic mutations using a variant of the heat diffusion approach. The obtained result indicates that there may be some evidence of co-location of pathogenicity-related proteins in the PPI networks. However, we have also found that substantial experiment-specific biases were a very prominent factor affecting the distribution of gene annotations in the network. Therefore, we conclude that many more gene annotations will be needed before this or similar approaches can reliably suggest candidates without the need of substantial expert input and follow-up curation. Out of the top 10 genes highlighted as likely important for pathogenicity using RWR approach, eight at present have not been adequately annotated. However, the remaining two genes have been annotated as an aspartokinase (FGRAMPH1_01T24779, top 4th prediction) and acetolactate synthase (FGRAMPH1_01T02707, top 6th prediction). Both genes have been previously identified as promising targets for antifungal agents in two earlier studies (Richie et al., 2013; Kaltdorf et al., 2016), respectively.

### Functional Cartography and Annotated PHI-Base Phenotypes

In an effort to describe the topological nature of the nodes that lie within the community structure detected in the first connected component of each network, a node classification scheme proposed by Guimera and Nunes Amaral (2005) has been employed. Here, we concentrate only on the first connected component of each network because it comprises the majority of the nodes of a given network and PHI-base annotated nodes mainly lie in the largest connected component of each network. The distribution of the node role types is recorded in Table 5. Overall, the majority of nodes within the community structure, calculated for the first connected component, are defined as non-hub peripheral nodes (R2) with most links within the community. Exception here is *Bipolaris sorokiniana* for which ultra-peripheral nodes (R1) account for the higher number within detected communities. On the other hand, hub-nodes (R5, R6, and R7) represent a very small percentage of the nodes across all networks.

**TABLE 5:** Functional cartography-specific node role distributions across all inferred interactomes.

**Species**	**R1 [%]**	**R2 [%]**	**R3 [%]**	**R4 [%]**	**R5 [%]**	**R6 [%]**	**R7 [%]**
*Aspergillus fumigatus*	29.411	49.218	16.806	4.092	0.255	0.182	0.036
*Bipolaris sorokiniana*	46.908	41.097	9.693	2.255	0.046	0.000	0.000
*Blumeria graminis* f. sp. *hordei*	19.439	55.514	17.444	7.280	0.000	0.243	0.081
*Botrytis cinerea*	28.511	54.924	13.063	2.944	0.355	0.169	0.034
*Colletotrichum fructicola*^1^	36.674	51.532	9.152	2.410	0.041	0.150	0.041
*Colletotrichum graminicola*	29.617	53.145	10.545	5.869	0.656	0.135	0.034
*Fusarium graminearum*	35.741	50.092	11.349	2.418	0.231	0.139	0.031
*Fusarium oxysporum* f. sp. *lycopersici*	36.930	50.812	8.995	3.117	0.000	0.119	0.026
*Fusarium verticillioides*	32.046	49.660	13.968	4.172	0.015	0.108	0.031
*Leptosphaeria maculans*	23.086	51.747	19.087	5.676	0.222	0.121	0.061
*Magnaporthe oryzae*	28.382	53.283	14.263	3.624	0.287	0.126	0.036
*Saccharomyces cerevisiae*	19.153	61.927	12.027	6.595	0.000	0.299	0.000
*Sclerotinia sclerotiorum*	30.926	51.742	13.141	3.993	0.000	0.170	0.028
*Verticillium dahliae*	29.440	55.017	10.678	4.676	0.000	0.170	0.019
*Zymoseptoria tritici*	25.356	49.904	19.377	5.190	0.000	0.115	0.058

*R1 – ultra-peripheral node (all links within the cluster), R2 – peripheral node (most links within the cluster), R3 – non-hub connector node (many links to other clusters), R4 – non-hub kinless node (links homogeneously spread among all clusters), R5 – provincial hub (hub node with majority links within its cluster), R6 – connector hub (hub with many links to other clusters), R7 – global kinless hub (hub with links homogeneously spread among all clusters); ^1^*Colletotrichum fructicola* previously known as *Colletotrichum gloeosporioides*.*

**FIGURE 4:**
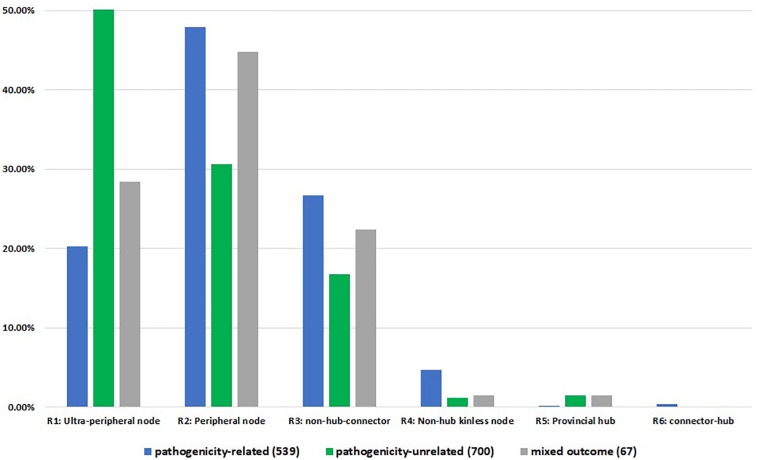
Node roles distribution according to PHI-base annotation. The numbers in brackets indicate the total number of annotated PHI-base phenotypes per largest connected component for 15 networks. R1 – ultra-peripheral node (all links within the cluster), R2 – peripheral node (most links within the cluster), R3 – non-hub connector node (many links to other clusters), R4 – non-hub kinless node (links homogeneously spread among all clusters), R5 – provincial hub (hub node with majority links within its cluster), R6 – connector hub (hub with many links to other clusters).

Whilst comparing the node associated phenotype to the node role, we identified 539 pathogenicity-related, 700 pathogenicity-unrelated and 67 with pathogenicity context specific phenotype nodes across first connected components of all networks (Figure 4). Pathogenicity-related nodes appeared to be highly represented by non-hub nodes, mainly peripheral nodes (R2) with the most links within the community. Although we observed connector hub nodes only associated with pathogenicity-related phenotype, the number is too small (2 nodes: FGRAMPH1_01T04861 and Sc YPL240C) to associate the R6 type nodes with pathogenicity. Unfortunately, the PHI-base annotation is not available for any of the global kinless hub nodes (R7). In total 28 nodes of this type were detected within the largest connected component of 13 PPI networks, whereas in *B. sorokiniana* and *S. cerevisiae* networks R7 nodes were not identified.

Furthermore, chi-square test of association confirmed initial findings that pathogenicity-related nodes are located outside the dense core of the network. The null hypothesis stating that there is no association between the node position in the network and its effect on the pathogenic lifestyle was rejected (χ^2^ = 127.97, critical value = 9.49, *p*-value = 1.0556E-26). Inspection of the frequency table (Supplementary Information S2) reveals that there is a positive correlation between node types R2, R3, and R4 and pathogenicity-related phenotypes. On the other hand, a significant positive correlation was observed between ultra-peripheral (R1) and pathogenicity-unrelated nodes.

Taken together, hub node genes were found in the majority to be unrelated to pathogenicity, while pathogenicity genes were overrepresented outside the core communities. In these peripheral regions the pathogenicity related genes link to one or more other communities. We also noted that pathogenicity related genes were not found in ultra-peripheral positions. Collectively these unexpected findings suggest that pathogenicity nodes join protein communities with diverse functions.

### Analysis of Small Interfering RNA Targets in Networks for *Botrytis cinerea* and *Fusarium graminearum*

To obtain additional information about the targeted proteins, protein complexes, and metabolic pathways and to determine the effectiveness of using the guilt-by-association principle (Petsko, 2009) in identifying associated candidate virulence genes, we investigated the protein–protein interaction neighbors of the 42 published siRNA target sites (Cai et al., 2018) identified in *B. cinerea* through wet biology/next generation sequencing analysis of the *in planta* interaction.

Both *B. cinerea* and *F. graminearum* are fungal Ascomycetes and many conserved orthologous genes exist in both species important for virulence on their respective hosts (Van De Wouw and Howlett, 2011). For *F. graminearum* a rich dataset of genes with phenotypic annotation exists, while for *B. cinerea* only a comparatively small number of genes have been formally tested in gene modification experiments and phenotypically assayed (Urban et al., 2017; Li et al., 2018). We reasoned that by surveying the predicted interactome of the siRNA target orthologs in *F. graminearum* additional information could be obtained to pinpoint siRNA targets to more specific protein complexes and metabolic networks, to provide further annotation to the interacting partners and to identify novel candidate genes with a potential function in virulence.

**FIGURE 5:**
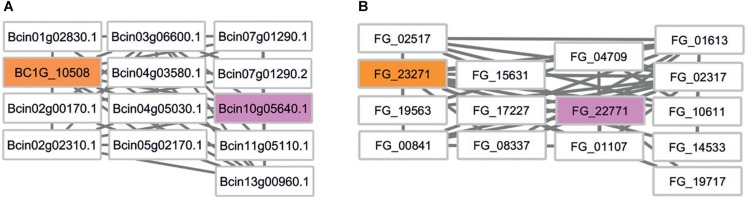
Comparative network analysis in *B. cinerea* and *F. graminearum*. **(A)** First-neighbor subnetwork of *B. cinerea* siRNA target BC1G_10508. Rectangular boxes depict nodes/gene identifiers. Colors indicate: orange – *B. cinerea* target, white – untested phenotype, pink – pathogenicity related phenotype in *F. graminearum*. **(B)** Comparative subnetwork from *F. graminearum.* The *B. cinerea* target ortholog is indicated in orange. FG_22771 encodes a pathogenicity related gene called *FgEB1* (PHI:7124).

We first mapped the siRNA targets identified in *B. cinerea* (Cai et al., 2018) to the *B. cinerea* and *F. graminearum* genomes using BLAST. This approach identified a total of 33 targets in the most recent *B. cinerea* genome assembly and 17 orthologs in *F. graminearum* (Supplementary Table S3). The siRNA target genes, the predicted interacting proteins and the phenotype annotation provided by PHI-base were then investigated using Cytoscape. Subnetworks of siRNA target genes and their first neighbors were created and visually inspected. In an attempt to keep functional annotation and the number of predicted candidate virulence genes small and meaningful, we set a stringent cut-off criterion requiring at least 1 in 10 genes to have a virulence associated annotation in the PHI-base database. Due to the lack of *B. cinerea* genes tested in gene function experiments, no *B. cinerea* target subnetwork fulfilled this stringent criterion. However, a *B. cinerea* subnetwork with one PHI-base virulence annotation in 13 genes exists and this is targeted by the small RNA TaAS1c-siR483 (Figure 5). The associated *F. graminearum* gene FG_22771 encodes the end-binding protein 1 (FgEb1) regulating microtubule dynamics. A deletion mutant of this gene shows increased hyphal branching and highly reduced sesquiterpene deoxynivalenol (DON) mycotoxin biosynthesis (Liu et al., 2017).

**FIGURE 6:**
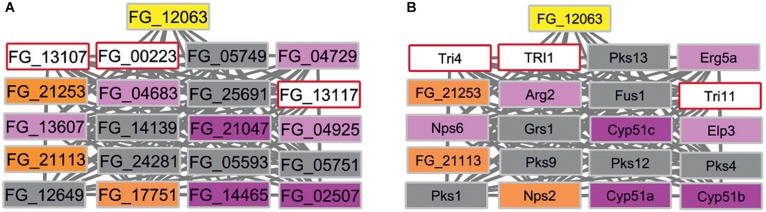
*F. graminearum* subnetwork containing three *B. cinerea* siRNA target homologs. **(A)** Three overlapping first-neighbor subnetworks contain three siRNA *B. cinerea* target gene orthologs (orange) and are connected to FG_12063 (yellow), independently identified as a wheat RNAi target. Nodes are colored to indicate target and phenotypes: orange (*B. cinerea* targets ortholog in *F. graminearum*), pink (pathogenicity related), magenta (mixed outcome where pathogen virulence is affected in some interactions but not others), gray (pathogenicity unrelated), white (unknown phenotype). **(B)** Same subnetwork displaying gene names taken from PHI-base instead of gene identifiers. Essential *CYP51* genes (magenta) and mycotoxin biosynthesis (pale blue) genes are identified within the network. Nps2 is a *B. cinerea* siRNA target ortholog and was shown to be pathogenicity related in some interactions.

In contrast, eight subnetworks in *F. graminearum* were identified that fulfilled the stringent cut-off criterion. The identified subnetworks have 4 to 89 node genes. We further excluded the largest subnetwork with 89 genes as this subnetwork includes many of the well-studied MAP kinase signaling related genes, i.e., *GPMK1*, *HOG1*, *MGV1* required for the virulence of *F. graminearum* and other fungal pathogens (Zhao et al., 2007). Subnetworks sharing first-neighbor genes were merged further (Supplementary Information S3). The candidate gene list includes seven *B. cinerea* target gene orthologs: FG_10451 is linked to Cdc42 implicated in cell division (Zhang et al., 2013); FG_03955 and FG_23275 are both linked to Hsp90 and Mgv1 with functions in heat shock and cell-wall integrity (Hou et al., 2002; Bui et al., 2016); FG_01625 is linked to the Top1 topoisomerase gene important for DNA unwinding and transcriptional regulation (Baldwin et al., 2010); FG_23313 is linked to two ATP driven efflux pumps Abc1 and Abc3 implicated in secretion of xenobiotics or to protect the fungus from host-derived defense compounds (Abou Ammar et al., 2013; Gardiner et al., 2013); FG_21253 and FG_21113 are linked to cytochrome P450 genes including cyp51 genes essential for ergosterol production required to maintain fungal plasma membrane integrity (Fan et al., 2013) and three cytochrome P450 monooxygenases involved in trichothecene mycotoxin production (Tri1, Tri4, Tri11) (Chen et al., 2019). An expected result was the linking of siRNA target homologs to genes involved in microtubule organization, stress adaptation, cell-wall integrity, DNA replication, and ATP driven efflux pumps because pathogens need to adapt to the many potentially hostile environments encountered during successful entry, colonization, and reproduction whilst exposed to the host’s defense responses. However, the identification of an additional subnetwork that included three ergosterol biosynthesis pathway genes (*CYP51)* as well as the secondary metabolism genes required for trichothecene mycotoxin production (*TRI1, TRI4, TRI11*) (Figure 6) was not expected. In various pathway databases, for example KEGG and MetaCyc, these pathways are displayed separately. This merged subnetwork included three target orthologs as first-neighbors and an additional single wheat siRNA target named FG_12063 reported to have an unknown MF, that was recently shown to be required for virulence (Jiao and Peng, 2018). For the subnetworks there are between one to six Pfam domains present in each protein forming the interactions. For example, the cytochrome P450 monooxygenase Tri1 has only one Pfam domain PF00067, whereas the polyketide synthase Pks1 has eight unique Pfam domains.

In summary for *F. graminearum*, the seven subnetworks obtained using this novel approach are formed by 69 genes, of which 36 have annotations provided by PHI-base or FusariumMutantDb. Thirty-five genes have not been experimentally analyzed previously in *F. graminearum* and have now been implicated as potential virulence factors. Our analysis suggests that many of these *F. graminearum* genes are involved in promoting stress adaptation, and that the corresponding *B. cinerea* genes may be involved in related metabolic functions. The potential link between the ergosterol biosynthesis pathway essential for fungal membrane formation and the secondary metabolism genes required for trichothecene mycotoxin production is a novel and unexpected finding.

### Network Availability

To facilitate access to these 15 interactomes, which we have called PHI-Nets, we have made them all available for download^4^. The use case example networks for *Fusarium graminearum* and *B. cinerea* were also uploaded to NDEx^5^ with accession numbers https://doi.org/10.18119/N9259J and https://doi.org/10.18119/N9XG68, respectively. Subnetworks can be found on NDEx using search term: PHI-Nets.

## Discussion

To fully understand biological mechanisms underlying complex processes such as fungal virulence and host invasion, functions of individual genes need to be considered in an appropriate context that can capture both their relationships to other biological entities and relevant system states. Biological networks have emerged as an important tool that enables large volumes of available information to be integrated and mined for such patterns. In this study we have created high-quality reconstructed interactomes for 14 species of pathogenic fungi and one model saprotroph across nine taxonomic orders within the Ascomycetes. Then by focusing on two exemplar species, we have illustrated how such resources can facilitate the identification of key interactions, reveal unexpected relationships in subnetworks annotated with PHI-base phenotype information and pinpoint possible candidate virulence genes with hitherto minimal to no formal annotation.

Unlike previous similar studies (Szklarczyk et al., 2019), a substantial component of our predicted networks was derived using DDI data, which can potentially allow the prediction of interactions even in cases where direct homology to known interacting proteins in other species cannot be established. Therefore, this approach may potentially offer more insights specifically for pathogenic fungal species where at present there are still very few experimentally confirmed interactions. The closest model organisms with well-profiled interactomes are the budding and fission yeasts (*S. cerevisiae* and *S. pombe*), which are not principally pathogenic and therefore are expected to be lacking many of the key genes and processes linked to virulence. Our evaluation of the interactome quality with respect to Gene Ontology function and cellular compartment annotations has shown that DDI-predicted edges are of comparable quality to interolog ones, and, likewise, are substantially better than random predictions. It should be noted that only 50% or less of the predicted exome can be captured within the protein–protein interaction network. Therefore, it was necessary to include interolog data to provide the more complete networks used in these analyses.

Notably, due to the differences in protein domain composition of the exomes some of the networks have considerable size differences despite having similar numbers of proteins. Though at present differences in the quality of the genome annotation cannot be fully discounted as a contributing factor, this may also hint at possible differences in organizational complexity of these organisms, as a greater number of interactions can accommodate a much larger range of emergent behaviors. Previous work has shown that the number of genes by itself does not correlate with an organism’s complexity, a phenomenon commonly referred to as ‘G-value paradox’ (Hahn and Wray, 2002). On the contrary, interactome size was shown to be one of the important determinants (Schad et al., 2011). Although this observation has not been further analyzed in detail in this study, the created resources may allow for future investigation of these patterns in pathogenic fungi. Similarly, although in each network the annotation for each node includes the predicted eight major cellular compartments, this information has not been explored beyond confirming co-localization of interacting partners.

We have investigated cartography analysis as a topological property in the network in the context of pathogenicity related and unrelated gene sets in 15 different fungal species. This analysis showed that genes important for pathogenicity appear to be located at the periphery of the densely connected network core, and in a relatively sparse area (lower within-community degree) compared to pathogenicity-unrelated genes. At the same time, genes important for pathogenicity were found to have higher participation coefficients. These two results were unexpected but are of considerable interest. These findings suggest their importance in mediating information flow through the network. In addition, 2 out of 10 genes highlighted in RWR analysis as ‘likely required for pathogenicity’ were found in peripheral region (R2) of the *F. graminearum* network indicating their non-hub like properties and links to other communities. Both genes were previously found to be required for virulence in a plant and a human pathogen and have been suggested as possible antifungal targets (Richie et al., 2013; Kaltdorf et al., 2016). Collectively, this outcome also suggests that as more phenotyping annotations become available via the PHI-base route, the knowledge available for these peripheral connected parts of the network, i.e., nodes located outside the dense core of the network, may disproportionately increase. Overtime this should reduce the length of candidate gene lists selected for follow-up functional analyses.

The main measurements of the topological properties of a network are node degree, betweenness centrality, average shortest path length and clustering coefficient. Studying these properties has been postponed until the PHI-annotations in the networks increase. Instead we have focused on node position in the network. In the protein–protein interaction network there is a topology where nodes with low degree (node with small number of edges connected to it) coexist with nodes with large degree (node with large number of edges connected to it). This also applies to the edge distributions in PPI networks where the density of edges within particular groups of nodes is higher than the average edge density in the whole network. Such groups of nodes with a high density of edges within them are defined as community structures (also known as modules or clusters). Each community consists of nodes that share similar properties or play a similar function in the graph. Thus, in protein–protein interaction networks, proteins that are within the same community are likely to share the same specific role within the cell (Fortunato, 2010). In our study, we identified pathogenicity-related nodes as non-hub peripheral nodes that have more links within the community (modules) they are part of. This indicates they share similar functions or even a similar pathogenic BP. However, these nodes also have some link to other functional modules (communities) which makes them important nodes in the network in mediating the information flow between different functional communities within the network. Thus, pathogenicity genes appear not to act alone but as a part of synergistic connections with other functional communities.

In contrast to the results by Liu et al. (2010) that compared pathogenicity-related genes to the rest of the network, our comparison was done with an experimentally confirmed pathogenicity-unrelated control gene set. The lower degree and location outside the dense core of the network are consistent with the expectation created by the currently adopted definition of pathogenicity-related genes (Idnurm and Howlett, 2001) as the ones that are only present in pathogenic species. Specifically, the core of the network would be composed of evolutionary older genes common to a much wider range of different species (Hahn and Wray, 2002). Additionally, gene deletion of vital core and high-degree genes are likely to be lethal to the organism and therefore would not produce an observable pathogenicity-related phenotype.

Although we have shown that properties of genes identified in this work appear to be predictive and therefore can be used to identify promising pathogenicity-related genes in diverse fungal species, limitations to this approach exist, in particular, the current availability of experimental phenotype data. As our approach relies on analysis of PPI networks to estimate the likely importance of genes both coverage and quality of such networks can be a limiting factor. At present and consistent with many previous studies our networks cover about half of all the genes in each species. Some important classes of infection-related proteins like effectors are unlikely to form interactions within the fungal cell. However, a further important factor is likely to be the current lack of experimentally determined interactions specific to pathogenic fungi. We estimate that once ∼33% of all genes for a single pathogenic species have been functionally characterized this will provide the ‘tipping point’ for this type of in-depth analysis via topological properties. Other potentially informative data sources we have not considered here are transcriptomics data and metabolic pathway networks. Transcriptomics has already been demonstrated to be informative in several previous studies but is often not available in sufficient quantities for some of the key fungal phytopathogenic species. In terms of the metabolic pathway networks, although they are unlikely to substantially improve coverage (as relatively few genes are enzymes), metabolic links between pathogen and host are of great importance and understanding these processes can help to identify promising candidate genes (Scharf et al., 2014; Dühring et al., 2015). Similarly, modeling of cross-species interactions between other types of host and pathogen networks is becoming an area of active research (Remmele et al., 2015; Guthke et al., 2016) that is likely to yield yet more insights to complement the inter-species interactomes constructed for this study. And lastly, as pathogenicity-related processes are highly context-specific, we expect that our results would be primarily useful in prioritization of promising candidates in combination with other gene lists that can provide appropriate context (for example, differential expression gene lists or relevant functional gene groups or chromosomal position).

Cross kingdom RNAi interference is an evolutionary conserved pathway in eukaryotes and plants. It can be utilized in crop protection strategies such as host-induced gene silencing and external small RNA applications to silence pathogen genes during infection (Majumdar et al., 2017; Mitter et al., 2017; Machado et al., 2018). In the two globally import pathosystems *B. cinerea*-tomato and *Fusarium*-wheat several studies demonstrated that both pathogen and host utilize RNA interference as part of pathogen virulence and host resistance mechanisms (Cai et al., 2018; Jiao and Peng, 2018). The presence of host-induced silencing mechanisms in wheat was previously demonstrated by expressing RNAi constructs targeting *F. graminearum* that resulted in attenuated virulence of the attacking *Fusarium* species (Chen et al., 2016). We used the 21 siRNA *B. cinerea* target genes published by Cai et al. (2018) to demonstrate that the PPI networks presented in this study can add further annotation to the targeted genes. The predicted direct protein interaction partners are more likely to have a function in virulence themselves and are therefore elevated to virulence gene candidate status. Due to the large numbers of proteins in the network, we focused our analysis on subnetworks in *F. graminearum* with a higher presence of PHI-base phenotypes to speculate on a potential role in virulence. A caveat to this approach is that using phenotype annotation from PHI-base is likely to introduce a bias as proteins with known annotation were preferentially selected to generate subnetworks. However, our approach identified 35 candidate virulence genes, including eight siRNA target gene orthologs themselves, that were mapped to RAS signaling, heat shock response, cell-wall integrity, ergosterol biosynthesis, trichothecene mycotoxin biosynthesis, DNA replication, and ATP driven export. The potential link found between ergosterol biosynthesis and trichothecene mycotoxin biosynthesis due to their co-occurrence within the same subnetwork is both intriguing and unexpected. Overall, these findings add further annotation to the siRNA targets previously identified (Cai et al., 2018), their unannotated potential interactors and map the *B. cinerea* siRNA targets to proteins targeted by azole fungicides in the wheat head blight pathogen *F. graminearum* (Fan et al., 2013). While *B. cinerea* is not a pathogen of wheat but of tomato and many other dicotyledonous hosts (Table 1), we suggest that the orthologous *B. cinerea* siRNA target genes in *F. graminearum* have a conserved function and may also likely be virulence genes in this species. While Cai et al. (2018) identified siRNAs from tomato, similar analysis are now underway in wheat. Recently FG_12063 encoding a protein with unknown function was suggested as the target of a small wheat RNA called Tae-miR1023 (Jiao and Peng, 2018). The deletion of FG_12063 reduced the pathogen’s ability to cause disease. The finding that FG_12063 is predicted to interact with the *B. cinerea* siRNA target homolog Nps2 identified in our *F. graminearum* subnetwork raises the possibility that siRNAs are also produced in wheat during defense against pathogen attack. Gene deletions of the prioritized genes presented in this work will be the focus of future investigations.

The projecting of the *B. cinerea* annotations arising from the RNA silenced targets onto the *F. graminearum* network yielded several unexpected results, that could not have been acquired solely through a straightforward pathway analysis. This is because in KEGG/MetaCyc pathways mostly enzymes are represented, whereas regulatory genes including kinases and transcription factors are not. In addition, pathway information is highly fragmented for filamentous pathogens. For instance, out of 13,447 *F. graminearum* proteins in the KEGG reference genome, 9,356 (70%) are currently not linked to any annotation or pathway. By using the network approach this allows researchers to overlay the pathways on the wider PPI network to permit the exploration of known pathways within a far richer context. For example, the cyp51 pathway is within the generic sterol biosynthesis pathway but through this PPI network analysis is also now linked by unknown mechanisms to additional genes not previously associated with sterol biosynthesis (including FG_12063, FG_21113, FG_21253) (as shown in Figure 6) and some of the genes responsible for trichothecence mycotoxin biosynthesis. In the original *Botrytis* study, the predicted siRNA target site had not been associated with sterol biosynthesis. Finally, for yeast model organisms excellent databases covering pathways, signaling and transcription factors annotations do exist; however, a different problem confronts their predictive use by molecular plant pathology/bioinformatics researchers. The overall size of the yeast proteome is considerably smaller (∼6,500) than for most filamentous pathogenic species (10,000–16,000). Therefore, large parts of PPI networks generated for filamentous pathogens do not correspond to any part of the PPI networks generated for these model non-pathogenic organisms.

This is the first study to explore the targets of small silencing RNAs delivered from host plants in the context of PPI networks for pathogenic species. This is also the first comparative study to explore whether new information on siRNA targeting obtained from one host–pathogen interaction can be used to provide novel insights for a second host–pathogen interaction which has already been extensively explored using traditional forward and reverse genetic approaches as well as through PPI network analysis.

The 15 PHI-Nets have been placed within the PHI-base resource. This will enable researchers to integrate novel phenotypes in a timely fashion to the networks/subnetworks of greatest interest. PHI-base entries are updated and extended 2–4 times a year. Also > 98% of PHI-base annotated proteins are mapped to Ensembl Genomes (Howe et al., 2019) and FungiDB browsers (Basenko et al., 2018), where RNA-seq data, variation data, and pathway maps for PHI-base proteins are available. This immediately provides researchers with an exciting and novel research environment within which to inter-connect and explore protein–protein relationships and pathways. In FungiDB release 46, subnetworks of interest for 8 of the 15 PHI-Net pathogen species (*A. fumigatus, B. cinerea, F. graminearum, F. oxysporum* f. sp. *lycopersici, F. verticillioides, M. oryzae, S. cerevisiae, S. sclerotiorum*) can also be mapped within FungiDB to KEGG and MetaCycDB pathways. In addition, Supplementary Table S2 (Col C-‘UniProt Id’ and Col E- ‘PHI-base mutant phenotype’) directly provides phenotypic annotation for proteins present in the 15 Ascomycete networks taken from PHI-base version 4.6. Here a corpus of UniProt Ids is provided rather than gene Ids. This information will directly assist researchers using a comparative genomics approach to identify species specific as well as conserved virulence functions across species and taxa. By using the data in this table researchers can more easily merge information provided by UniProtKB (GO information, subcellular location, enzymatic activity) with the in-host phenotypes provided by PHI-base. Finally, PHI-base already provides detailed biological lifestyle information for PHI-base species to allow non-specialist researchers easy access to pathogen information to enable comparative studies (obligate biotrophs, heterotrophic and necrotrophic lifestyles) (Table 1) and published previously (Urban et al., 2015). The use case example networks and subnetworks for *F. graminearum* and *B. cinerea* were further uploaded to NDEx (see footnote 5) to increase visibility of this study for wet lab molecular biologists and bioinformaticians alike. NDEx provides a rich infrastructure for network access and is closely linked to Cytoscape and promotes re-use of research findings (Pratt et al., 2015; Pillich et al., 2017). NDEx also enables programmatic access via APIs and can be used to embed subnetworks directly into webpages (Pratt et al., 2015; Pillich et al., 2017).

## Conclusion

We provide predicted protein–protein interaction networks of globally important filamentous plant pathogens for download and interactively accessible online versions at the network repository PHI-Nets^6^ and NDEx (see footnote 5). We have also identified a set of features that can be effectively used to identify candidate virulence and pathogenicity genes in pathogenic fungi. Exemplar networks for *B. cinerea* and *F. graminearum* were used to enrich annotation for several *B. cinerea* genes targeted by small interfering RNAs produced by the *Arabidopsis* host during disease interaction. Several directly interacting proteins of the target genes were identified and are novel candidate virulence genes in both *B. cinerea* and *F. graminearum.* We predict that as more genomes are sequenced, and more pathogen genes are functionally characterized this will result in a data increase in interactome databases. Thus, networks will need to be rebuilt over time to take these latest developments into consideration when exploring strain-to-strain differences in pangenome and/or genome wide association studies. We also predict that once more protein–protein interactions are experimentally verified for pathogenic species, these can be used to increase the robustness and extend of DDI networks, permit topological properties of a network to be explored in detail and thereby increase their overall utility to comparative analyses when exploring host–pathogen and pathogen–pathogen interactions.

## Data Availability Statement

The datasets generated for this study can be found in the Pathogen–Host interaction database portal http://www.phi-base.org/consortium.htm. The use case example networks for *Fusarium graminearum* and *Botrytis cinerea* were further uploaded to NDEx (www.ndexbio.org). Computational scripts were made available at https://github.com/PHI-base/phi-nets/.

## Author Contributions

EJ-S and AL: initial ideas, bioinformatic analysis, and manuscript writing. MU: initial ideas, visualization of networks in Cytoscape, biology, and manuscript writing. ST and CR: drafting manuscript and comments. KH-K: initial ideas, manuscript writing, data analysis, and biology. All authors read and approved the final manuscript.

## Conflict of Interest

The authors declare that the research was conducted in the absence of any commercial or financial relationships that could be construed as a potential conflict of interest.

## Footnotes

1 www.PHI-base.org

2 ftp://ftp.ensemblgenomes.org/pub/fungi/

3 http://fungi.ensembl.org

4 www.PHI-base.org

5 www.ndexbio.org

6 www.phi-base.org/consortium.htm

## Supplementary Material

TABLE S1 Genome information for 15 selected species.

TABLE S2 Node annotation table for all networks.

TABLE S3 *Botrytis cinerea* and *F. graminearum* nearest neighbor subnetworks containing siRNA target genes or orthologs.

INFORMATION S1 Solving domains overlapping – manual approach.

INFORMATION S2 Cartography analysis and Chi-square test of association.

INFORMATION S3 Additional graphical display of *B. cinerea* and *F. graminearum* nearest neighbor subnetworks.

## References

[B1] Abou Ammar G. Tryono R. Doll K. Karlovsky P. Deising H. B. Wirsel S. G. (2013). Identification of ABC transporter genes of *Fusarium graminearum* with roles in azole tolerance and/or virulence. *PLoS One* 8:e79042. 10.1371/journal.pone.0079042 24244413 PMC3823976

[B2] Ashburner M. Ball C. A. Blake J. A. Botstein D. Butler H. Cherry J. M. (2000). Gene ontology: tool for the unification of biology. *Nat. Genet.* 25 25–29. 10802651 10.1038/75556 PMC3037419

[B3] Baldwin T. K. Urban M. Brown N. Hammond-Kosack K. E. (2010). A role for topoisomerase I in *Fusarium graminearum* and *F. culmorum* pathogenesis and sporulation. *Mol. Plant Microbe Interact.* 23 566–577. 10.1094/MPMI-23-5-0566 20367465

[B4] Baldwin T. T. Basenko E. Harb O. Brown N. A. Urban M. Hammond-Kosack K. E. (2018). Sharing mutants and experimental information prepublication using FgMutantDb. *Fungal Genet. Biol.* 115 90–93. 10.1016/j.fgb.2018.01.002 29355605

[B5] Basenko E. Y. Pulman J. A. Shanmugasundram A. Harb O. S. Crouch K. Starns D. (2018). FungiDB: an integrated bioinformatic resource for fungi and oomycetes. *J. Fungi.* 4:E39. 10.3390/jof4010039 30152809 PMC5872342

[B6] Bennett L. Lysenko A. Papageorgiou L. G. Urban M. Hammond-Kosack K. Rawlings C. (2012). “Detection of multi-clustered genes and community structure for the plant pathogenic fungus *Fusarium graminearum*,” in *Proceedings of the 10th International Conference on Computational Methods in Systems Biology*, London, 69–86. 10.1007/978-3-642-33636-2_6

[B7] Blondel V. D. Guillaume J.-L. Lambiotte R. Lefebvre E. (2008). Fast unfolding of communities in large networks. *J. Stat. Mech.* 2008:10008. 21517554

[B8] Bui D. C. Lee Y. Lim J. Y. Fu M. Kim J. C. Choi G. J. (2016). Heat shock protein 90 is required for sexual and asexual development, virulence, and heat shock response in *Fusarium graminearum*. *Sci. Rep.* 6:28154. 10.1038/srep28154 27306495 PMC4910114

[B9] Cai Q. Qiao L. Wang M. He B. Lin F. M. Palmquist J. (2018). Plants send small RNAs in extracellular vesicles to fungal pathogen to silence virulence genes. *Science* 360 1126–1129. 10.1126/science.aar4142 29773668 PMC6442475

[B10] Cairns T. C. Studholme D. J. Talbot N. J. Haynes K. (2016). New and improved techniques for the study of pathogenic fungi. *Trends Microbiol.* 24 35–50. 10.1016/j.tim.2015.09.008 26549580

[B11] Chen W. Kastner C. Nowara D. Oliveira-Garcia E. Rutten T. Zhao Y. (2016). Host-induced silencing of *Fusarium culmorum* genes protects wheat from infection. *J. Exp. Bot.* 67 4979–4991. 10.1093/jxb/erw263 27540093 PMC5014151

[B12] Chen Y. Kistler H. C. Ma Z. (2019). *Fusarium graminearum* trichothecene mycotoxins: biosynthesis, regulation, and management. *Annu. Rev. Phytopathol.* 57 15–39. 10.1146/annurev-phyto-082718-100318 30893009

[B13] Dean R. Van Kan J. A. Pretorius Z. A. Hammond-Kosack K. E. Di Pietro A. Spanu P. D. (2012). The top 10 fungal pathogens in molecular plant pathology. *Mol. Plant Pathol.* 13 414–430. 10.1111/j.1364-3703.2011.00783.x 22471698 PMC6638784

[B14] Dühring S. Germerodt S. Skerka C. Zipfel P. F. Dandekar T. Schuster S. (2015). Host-pathogen interactions between the human innate immune system and *Candida albicans*—understanding and modeling defense and evasion strategies. *Front. Microbiol.* 6:625. 10.3389/fmicb.2015.00625 26175718 PMC4485224

[B15] Eddy S. R. (2009). A new generation of homology search tools based on probabilistic inference. *Genome Inform.* 23 205–211. 20180275

[B16] Ellison C. E. Kowbel D. Glass N. L. Taylor J. W. Brem R. B. (2014). Discovering functions of unannotated genes from a transcriptome survey of wild fungal isolates. *mBio* 5:e1046-13. 10.1128/mBio.01046-13 24692637 PMC3977361

[B17] Fan J. Urban M. Parker J. E. Brewer H. C. Kelly S. L. Hammond-Kosack K. E. (2013). Characterization of the sterol 14alpha-demethylases of *Fusarium graminearum* identifies a novel genus-specific CYP51 function. *New Phytol.* 198 821–835. 10.1111/nph.12193 23442154

[B18] Finn R. D. Coggill P. Eberhardt R. Y. Eddy S. R. Mistry J. Mitchell A. L. (2016). The Pfam protein families database: towards a more sustainable future. *Nucleic Acids Res.* 44 D279–D285. 10.1093/nar/gkv1344 26673716 PMC4702930

[B19] Fortunato S. (2010). Community detection in graphs. *Phys. Rep. Rev. Sec. Phys. Lett.* 486 75–174. 10.1016/j.physrep.2009.11.002

[B20] Gardiner D. M. Stephens A. E. Munn A. L. Manners J. M. (2013). An ABC pleiotropic drug resistance transporter of *Fusarium graminearum* with a role in crown and root diseases of wheat. *FEMS Microbiol. Lett.* 348 36–45. 10.1111/1574-6968.12240 23965171

[B21] Ghoorah A. W. Devignes M. D. Smail-Tabbone M. Ritchie D. W. (2014). KBDOCK 2013: a spatial classification of 3D protein domain family interactions. *Nucleic Acids Res.* 42 D389–D395. 10.1093/nar/gkt1199 24271397 PMC3964971

[B22] Guimera R. Nunes Amaral L. A. (2005). Functional cartography of complex metabolic networks. *Nature* 433 895–900. 10.1038/nature03288 15729348 PMC2175124

[B23] Guthke R. Gerber S. Conrad T. Vlaic S. Durmus S. Cakir T. (2016). Data-based reconstruction of gene regulatory networks of fungal pathogens. *Front. Microbiol.* 7:570. 10.3389/fmicb.2016.00570 27148247 PMC4840211

[B24] Hahn M. W. Wray G. A. (2002). The g-value paradox. *Evol. Dev.* 4 73–75. 12004964 10.1046/j.1525-142x.2002.01069.x

[B25] He F. Zhang Y. Chen H. Zhang Z. Peng Y.-L. (2008). The prediction of protein-protein interaction networks in rice blast fungus. *BMC Genomics* 9:519. 10.1186/1471-2164-9-519 18976500 PMC2601049

[B26] Herrero J. Muffato M. Beal K. Fitzgerald S. Gordon L. Pignatelli M. (2016). Ensembl comparative genomics resources. *Database* 2016:bav096. 10.1093/database/bav096 26896847 PMC4761110

[B27] Hou Z. M. Xue C. Y. Peng Y. L. Katan T. Kistler H. C. Xu J. R. (2002). A mitogen-activated protein kinase gene (MGV1) in *Fusarium graminearum* is required for female fertility, heterokaryon formation, and plant infection. *Mol. Plant Microbe Interact.* 15 1119–1127. 10.1094/mpmi.2002.15.11.1119 12423017

[B28] Howe K. L. Contreras-Moreira B. De silva N. Maslen G. Akanni W. Allen J. (2019). Ensembl Genomes 2020—enabling non-vertebrate genomic research. *Nucleic Acids Res.* 2019:gkz890. 10.1093/nar/gkz890 31598706 PMC6943047

[B29] Idnurm A. Howlett B. J. (2001). Pathogenicity genes of phytopathogenic fungi. *Mol. Plant Pathol.* 2 241–255. 10.1046/j.1464-6722.2001.00070.x 20573012

[B30] Jiao J. Peng D. (2018). Wheat microRNA1023 suppresses invasion of *Fusarium graminearum* via targeting and silencing FGSG_03101. *J. Plant Interact.* 13 514–521. 10.1080/17429145.2018.1528512

[B31] Kaltdorf M. Srivastava M. Gupta S. K. Liang C. Binder J. Dietl A. M. (2016). Systematic identification of anti-fungal drug targets by a metabolic network approach. *Front. Mol. Biosci.* 3:22. 10.3389/fmolb.2016.00022 27379244 PMC4911368

[B32] Kersey P. J. Allen J. E. Allot A. Barba M. Boddu S. Bolt B. J. (2018). Ensembl genomes 2018: an integrated omics infrastructure for non-vertebrate species. *Nucleic Acids Res.* 46 D802–D808. 10.1093/nar/gkx1011 29092050 PMC5753204

[B33] Kersey P. J. Allen J. E. Armean I. Boddu S. Bolt B. J. Carvalho-Silva D. (2016). Ensembl genomes 2016: more genomes, more complexity. *Nucleic Acids Res.* 44 D574–D580. 10.1093/nar/gkv1209 26578574 PMC4702859

[B34] Kim H. Jung K. W. Maeng S. Chen Y. L. Shin J. Shim J. E. (2015). Network-assisted genetic dissection of pathogenicity and drug resistance in the opportunistic human pathogenic fungus *Cryptococcus neoformans*. *Sci. Rep.* 5:8767. 10.1038/srep08767 25739925 PMC4350084

[B35] Kim M. Zhang H. Woloshuk C. Shim W.-B. Yoon B.-J. (2015). Computational prediction of pathogenic network modules in *Fusarium verticillioides*. *IEEE/ACM Trans. Comput. Biol. Bioinform.* 15 506–515. 10.1109/TCBB.2015.2440232 29610099

[B36] Kinsella R. J. Kahari A. Haider S. Zamora J. Proctor G. Spudich G. (2011). Ensembl BioMarts: a hub for data retrieval across taxonomic space. *Database* 2011:bar030. 10.1093/database/bar030 21785142 PMC3170168

[B37] Köhler S. Bauer S. Horn D. Robinson P. N. (2008). Walking the interactome for prioritization of candidate disease genes. *Am. J. Hum. Genet.* 82 949–958. 10.1016/j.ajhg.2008.02.013 18371930 PMC2427257

[B38] Lei D. Lin R. Yin C. Li P. Zheng A. (2014). Global protein–protein interaction network of rice sheath blight pathogen. *J. Proteome Res.* 13 3277–3293. 10.1021/pr500069r 24894516

[B39] Leiserson M. D. Vandin F. Wu H. T. Dobson J. R. Eldridge J. V. Thomas J. L. (2015). Pan-cancer network analysis identifies combinations of rare somatic mutations across pathways and protein complexes. *Nat. Genet.* 47 106–114. 10.1038/ng.3168 25501392 PMC4444046

[B40] Li H. Zhang Z. D. (2016). Systems understanding of plant-pathogen interactions through genome-wide protein-protein interaction networks. *Front. Agric. Sci. Eng.* 3:102–112. 10.15302/J-Fase-2016100

[B41] Li S. Musungu B. Lightfoot D. Ji P. (2018). The interactomic analysis reveals pathogenic protein networks in *Phomopsis longicolla* underlying seed decay of soybean. *Front. Genet.* 9:104. 10.3389/fgene.2018.00104 29666630 PMC5891612

[B42] Liu X. Tang W.-H. Zhao X.-M. Chen L. (2010). A network approach to predict pathogenic genes for *Fusarium graminearum*. *PLoS One* 5:e13021. 10.1371/journal.pone.0013021 20957229 PMC2949387

[B43] Liu Z. Wu S. Chen Y. Han X. Gu Q. Yin Y. (2017). The microtubule end-binding protein FgEB1 regulates polar growth and fungicide sensitivity via different interactors in *Fusarium graminearum*. *Environ. Microbiol.* 19 1791–1807. 10.1111/1462-2920.13651 28028881

[B44] Lord P. W. Stevens R. D. Brass A. Goble C. A. (2003). Investigating semantic similarity measures across the gene ontology: the relationship between sequence and annotation. *Bioinformatics* 19 1275–1283. 10.1093/bioinformatics/btg153 12835272

[B45] Lysenko A. Urban M. Bennett L. Tsoka S. Janowska-Sejda E. Rawlings C. J. (2013). Network-based data integration for selecting candidate virulence associated proteins in the cereal infecting fungus *Fusarium graminearum*. *PLoS One* 8:e67926. 10.1371/journal.pone.0067926 23861834 PMC3701590

[B46] Machado A. K. Brown N. A. Urban M. Kanyuka K. Hammond-Kosack K. E. (2018). RNAi as an emerging approach to control *Fusarium* head blight disease and mycotoxin contamination in cereals. *Pest. Manag. Sci.* 74 790–799. 10.1002/ps.4748 28967180 PMC5873435

[B47] Majumdar R. Rajasekaran K. Cary J. W. (2017). RNA interference (RNAi) as a potential tool for control of mycotoxin contamination in crop plants: concepts and considerations. *Front. Plant Sci.* 8:200. 10.3389/fpls.2017.00200 28261252 PMC5306134

[B48] Mitter N. Worrall E. A. Robinson K. E. Li P. Jain R. G. Taochy C. (2017). Clay nanosheets for topical delivery of RNAi for sustained protection against plant viruses. *Nat. Plants* 3:16207. 10.1038/nplants.2016.207 28067898

[B49] Orchard S. Ammari M. Aranda B. Breuza L. Briganti L. Broackes-Carter F. (2014). The MIntAct project–IntAct as a common curation platform for 11 molecular interaction databases. *Nucleic Acids Res.* 42 D358–D363. 10.1093/nar/gkt1115 24234451 PMC3965093

[B50] Petsko G. A. (2009). Guilt by association. *Genome Biol.* 10:104. 10.1186/gb-2009-10-4-104 19439034 PMC2688918

[B51] Pillich R. T. Chen J. Rynkov V. Welker D. Pratt D. (2017). NDEx: a community resource for sharing and publishing of biological networks. *Methods Mol. Biol.* 1558 271–301. 10.1007/978-1-4939-6783-4_13 28150243

[B52] Pratt D. Chen J. Welker D. Rivas R. Pillich R. Rynkov V. (2015). NDEx, the network data exchange. *Cell Syst.* 1 302–305. 10.1016/j.cels.2015.10.001 26594663 PMC4649937

[B53] Raffaele S. Kamoun S. (2012). Genome evolution in filamentous plant pathogens: why bigger can be better. *Nat. Rev. Microbiol.* 10 417–430. 10.1038/nrmicro2790 22565130

[B54] Remmele C. W. Luther C. H. Balkenhol J. Dandekar T. Müller T. Dittrich M. T. (2015). Integrated inference and evaluation of host–fungi interaction networks. *Front. Microbiol.* 6:764. 10.3389/fmicb.2015.00764 26300851 PMC4523839

[B55] Richie D. L. Thompson K. V. Studer C. Prindle V. C. Aust T. Riedl R. (2013). Identification and evaluation of novel acetolactate synthase inhibitors as antifungal agents. *Antimicrob. Agents Chemother.* 57 2272–2280. 10.1128/AAC.01809-12 23478965 PMC3632958

[B56] Schad E. Tompa P. Hegyi H. (2011). The relationship between proteome size, structural disorder and organism complexity. *Genome Biol.* 12:R120. 10.1186/gb-2011-12-12-r120 22182830 PMC3334615

[B57] Scharf D. H. Heinekamp T. Brakhage A. A. (2014). Human and plant fungal pathogens: the role of secondary metabolites. *PLoS Pathog.* 10:e1003859. 10.1371/journal.ppat.1003859 24497825 PMC3907374

[B58] Seidl M. F. Van Den Ackerveken G. Govers F. Snel B. (2011). A domain-centric analysis of oomycete plant pathogen genomes reveals unique protein organization. *Plant Physiol.* 155 628–644. 10.1104/pp.110.167841 21119047 PMC3032455

[B59] Seyedmousavi S. Guillot J. Arne P. De Hoog G. S. Mouton J. W. Melchers W. J. (2015). *Aspergillus* and *aspergilloses* in wild and domestic animals: a global health concern with parallels to human disease. *Med. Mycol.* 53 765–797. 10.1093/mmy/myv067 26316211

[B60] Smedley D. Kohler S. Czeschik J. C. Amberger J. Bocchini C. Hamosh A. (2014). Walking the interactome for candidate prioritization in exome sequencing studies of Mendelian diseases. *Bioinformatics* 30 3215–3222. 10.1093/bioinformatics/btu508 25078397 PMC4221119

[B61] Spanu P. D. Abbott J. C. Amselem J. Burgis T. A. Soanes D. M. Stuber K. (2010). Genome expansion and gene loss in powdery mildew fungi reveal tradeoffs in extreme parasitism. *Science* 330 1543–1546. 10.1126/science.1194573 21148392

[B62] Stein A. Russell R. B. Aloy P. (2005). 3did: interacting protein domains of known three-dimensional structure. *Nucleic Acids Res.* 33 D413–D417. 15608228 10.1093/nar/gki037 PMC539991

[B63] Szklarczyk D. Gable A. L. Lyon D. Junge A. Wyder S. Huerta-Cepas J. (2019). STRING v11: protein-protein association networks with increased coverage, supporting functional discovery in genome-wide experimental datasets. *Nucleic Acids Res.* 47 D607–D613. 10.1093/nar/gky1131 30476243 PMC6323986

[B64] Urban M. Cuzick A. Rutherford K. Irvine A. Pedro H. Pant R. (2017). PHI-base: a new interface and further additions for the multi-species pathogen–host interactions database. *Nucleic Acids Res.* 45 D604–D610. 10.1093/nar/gkw1089 27915230 PMC5210566

[B65] Urban M. Pant R. Raghunath A. Irvine A. G. Pedro H. Hammond-Kosack K. E. (2015). The pathogen-host interactions database (PHI-base): additions and future developments. *Nucleic Acids Res.* 43 D645–D655. 10.1093/nar/gku1165 25414340 PMC4383963

[B66] Van De Wouw A. P. Howlett B. J. (2011). Fungal pathogenicity genes in the age of ‘omics’. *Mol. Plant Pathol.* 12 507–514. 10.1111/j.1364-3703.2010.00680.x 21535355 PMC6640419

[B67] Wang T. Y. He F. Hu Q. W. Zhang Z. (2011). A predicted protein-protein interaction network of the filamentous fungus *Neurospora crassa*. *Mol. Biosyst.* 7 2278–2285. 10.1039/c1mb05028a 21584303

[B68] Weiberg A. Jin H. (2015). Small RNAs–the secret agents in the plant-pathogen interactions. *Curr. Opin. Plant Biol.* 26 87–94. 10.1016/j.pbi.2015.05.033 26123395 PMC4573252

[B69] Weiberg A. Wang M. Lin F. M. Zhao H. W. Zhang Z. H. Kaloshian I. (2013). Fungal small RNAs suppress plant immunity by hijacking host RNA interference pathways. *Science* 342 118–123. 10.1126/science.1239705 24092744 PMC4096153

[B70] Yellaboina S. Tasneem A. Zaykin D. V. Raghavachari B. Jothi R. (2011). DOMINE: a comprehensive collection of known and predicted domain-domain interactions. *Nucleic Acids Res.* 39 D730–D735. 10.1093/nar/gkq1229 21113022 PMC3013741

[B71] Zhang C. Wang Y. Wang J. Zhai Z. Zhang L. Zheng W. (2013). Functional characterization of Rho family small GTPases in *Fusarium graminearum*. *Fungal Genet Biol.* 61 90–99. 10.1016/j.fgb.2013.09.001 24055721

[B72] Zhao X. Mehrabi R. Xu J.-R. (2007). Mitogen-activated protein kinase pathways and fungal pathogenesis. *Eukaryot. Cell* 6 1701–1714. 10.1128/ec.00216-07 17715363 PMC2043402

[B73] Zhao X. M. Zhang X. W. Tang W. H. Chen L. (2009). FPPI: *Fusarium graminearum* protein-protein interaction database. *J. Proteome Res.* 8 4714–4721. 10.1021/pr900415b 19673500

