# Supplementary material for: PHI-Nets: A Network Resource for Ascomycete Fungal Pathogens to Annotate and Identify Putative Virulence Interacting Proteins and siRNA Targets
Source: Front Microbiol. 2019 Dec 6;10:2721. doi: 10.3389/fmicb.2019.02721 (PMC6908471; doi:10.3389/fmicb.2019.02721)
Supplement: INFORMATION S1 — Solving domains overlapping – manual approach. [file Data_Sheet_1.pdf]

Supplementary information 1

Solving domains overlapping – manual approach

Firstly, the score for each domain is taken into account. If the score is equal for all or some of the domains, the pairwise score comparison for those is performed. If the pairwise score comparison is not able to resolve the overlapping (is not able to point out the best domain) the domain with the best e-value (from HMMER 3 run) is chosen from the group of domains with the same score and the same results of pairwise score comparison. The example of scoring table together with the information generated with the aid of HMMER 3 run is presented in Tables 1 A and B.

1. Steps in finding the best solution for overlapping domains in the example from the Table 1.

a. All of domains overlap with each other (Table 1 A, column c and d). The best equal score (1 x 0, 4 x 1) was calculated for three domains: PF08241, PF13847 and PF13489 (Table 1 B). The next step is to perform pairwise comparison of these three domains.

b. Pairwise score comparison of 3 domains from point 1:

- Comparison of domain 1 and domain 3 results in score equals to 0 (row 1, column 3) – exclusion of domain 1
- Comparison of domain 3 and domain 1 results in score equals to 1 (row 3, column 1) –domain 3 has a chance to be the best one
- Comparison of domain 6 and domain 3 results in score equals to 1 (row 6, column 3) – exclusion of domain 3 and chance for domain 6 to be the best one.
- Comparison of domain 6 and domain 1 results in score equals to 0 (row 6, column 1)

Thus, overlapping is not resolved via pairwise score comparison. Then, the domain (from these three domains with the same score) with the best e-value is chosen. In the example above domain PF08241 was chosen as the best one.

c. The last step: the domain with the lowest e-value (HMMER run) is chosen. In the above example the domain PF08241 has the lowest e-value. (Table 1 A, columns g and h). Therefore, domain PF08241 is chosen.

Table 1 Example of finding the best domain based first on the score, then the pairwise score and finally on the best e-value.

A. The information from HMMER run

a	b	c	d	e	f	g	h
Pfam ID	Domain description	Start	End	End -Start	Score	e-value	-Log10 (e-value)
PF08241	Methyltransferase domain	40	146	106	55.48	3.00E-13	12.5229
PF08242	Methyltransferase domain	40	144	104	49.24	2.20E-11	10.6576
PF13847	Methyltransferase domain	33	196	163	40.11	1.20E-08	7.9208
PF13649	Methyltransferase domain	39	142	103	34.17	7.70E-07	6.1135
PF12847	Methyltransferase domain	35	149	114	34.07	8.20E-07	6.0862
PF13489	Methyltransferase domain	8	202	194	32.75	0.000002	5.699

B. Scores: (-1) – domain does not overlap, (1) – domain overlaps and it is the best choice (win), (0) – domain overlaps and is not taken into account.

No	Pfam ID	PF08241	PF08242	PF13847	PF13649	PF12847	PF13489
1	PF08241	-1	1	0	1	1	1
2	PF08242	0	-1	0	1	0	0
3	PF13847	1	1	-1	1	1	0
4	PF13649	0	0	0	-1	0	0
5	PF12847	0	1	0	1	-1	0
6	PF13489	0	1	1	1	1	-1
