# Supplementary material for: PHI-Nets: A Network Resource for Ascomycete Fungal Pathogens to Annotate and Identify Putative Virulence Interacting Proteins and siRNA Targets
Source: Front Microbiol. 2019 Dec 6;10:2721. doi: 10.3389/fmicb.2019.02721 (PMC6908471; doi:10.3389/fmicb.2019.02721)
Supplement: INFORMATION S2 — Cartography analysis and Chi-square test of association. [file Data_Sheet_2.pdf]

Supplementary information 2

Community structure definition

Complex networks such as biological systems like protein-protein interaction networks have a topology where nodes with low degree coexist with nodes with large degree. This also applies to the edge distributions in the networks where the density of edges within particular groups of nodes is higher than the average edge density in the whole network. Such groups of nodes with a high density of edges within them are defined as community structures (also known as modules or clusters) (Fortunato, 2010, Fortunato and Castellano, 2012), see Figure 1 reproduced from Fortunato and Castellano (2012). Each community consists of nodes that share similar properties or play a similar function in the graph. Thus, in protein-protein interaction networks proteins that are within the same community are likely to share the same specific role within the cell (Fortunato, 2010).

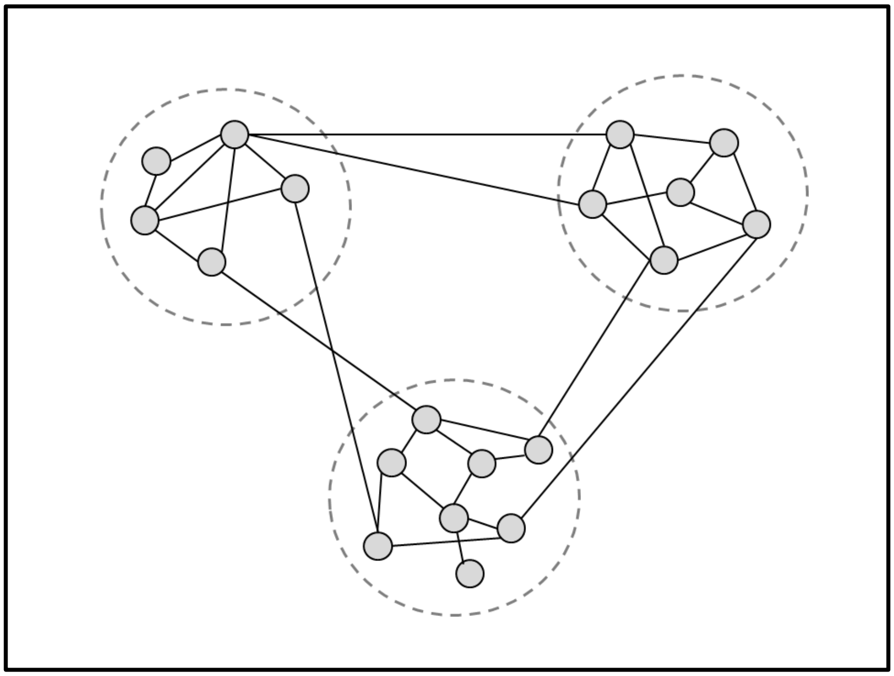

Figure 1 A simple graph with three communities.
Nodes and links are respectively depicted by circles and straight lines. Each dashed circle represents community structure (module).

Community structure detection

The community structure of the main connected component was identified by means of the greedy agglomerative algorithm knows as Louvain method (Blondel et al., 2008), Figure 2. The method has the advantage of being very fast and accurate despite its greedy nature. The algorithm consists of two main phases: a modularity optimisation and a community aggregation. In the first phase, each node represents an individual module. Then, successive iteration takes place over all nodes to verify which vertices should be connected to increase the modularity. The process is repeated until no further improvement in the modularity can be obtained. In the second phase of the algorithm, a new network is formed where the communities that have been established in the first phase become nodes in the new network and the links between those nodes are given the weight which is a sum of the weights of the links that join the two corresponding communities. Also, links between nodes in the same community become self-loops for this community in the new network. The step is repeated until no further gain in the modularity is achievable. As a result, it is possible to attain the best partitions of the initial network into communities.

Thus, looking at the network example depicted in Figure 2 (Blondel et al., 2008), 13 nodes (illustrated in light blue colour) represent 13 communities in the initial network. After modular optimisation and community aggregation, a new network of four nodes (green, blue, red, and light blue) is created. Then, both phases of the algorithm are repeated on the created network of four nodes. As consequence of the second pass of the algorithm, green and blue nodes fall into one community. Similarly, the red and light blue nodes becoming part of the second community of the third network. This is because a weight on the link between green and blue node is higher (4) comparing to a weight on the link between green and other two nodes (1), just as a weight on the link between red and light blue node (3) comparing to a weight on the link between red and green nodes (1). Thus, the blue node becomes a part of green node community and red node becomes a part of light blue node community leading to a weighted link between newly created communities equal to 3 (summary of links between green and light blue nodes, green and red nodes, and blue and light blue nodes). Finally, no further improvement in the modularity of a newly created third network could be obtained.

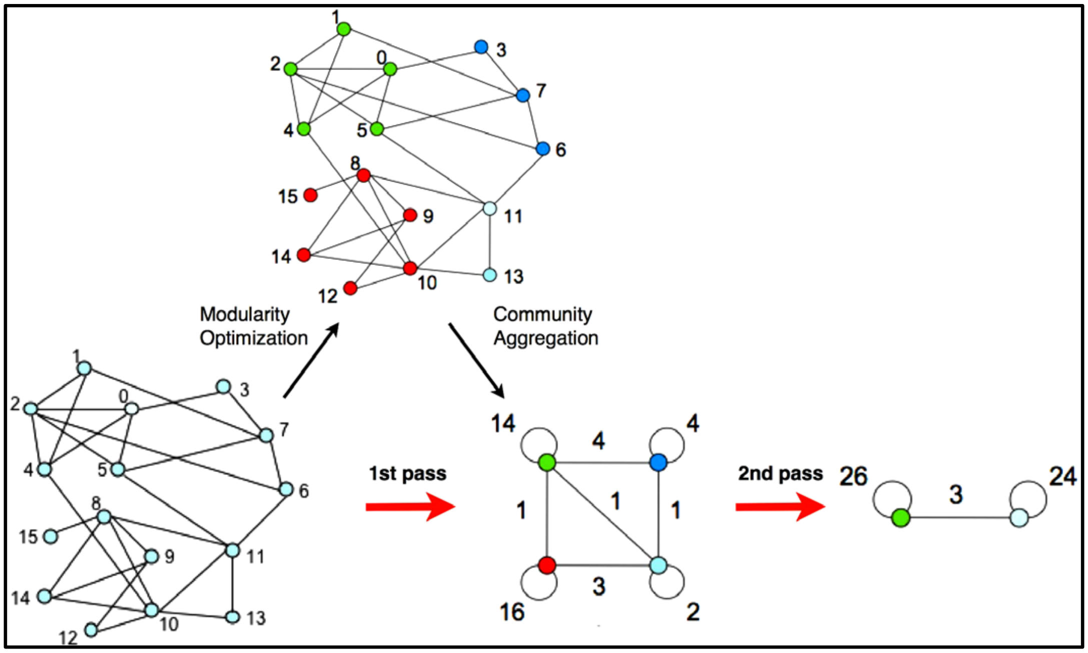

Figure 2 Visualisation of the steps of Louvain algorithm.
Where red, blue, green and light blue nodes indicate four different communities (modules). Weights of links between new nodes are the sum of the weight of the links between nodes in the corresponding two communities. Weight on new nodes is the number of self-loops calculated as links between nodes in the same community. For example links for the blue community: L={(3,7), (7,3), (7,6), (6,7)} account for 4 self-loops after 1st pass of the algorithm (Image copied from Blondel et al., 2008).

Node role assignment – cartography analysis

A node role is characterised according to two measures adopted from the Guimera and Nunes Amaral (2005) study: within-community degree z-**score** and participation coefficient P. **Degree z-score** measures the connectivity of the node to members of the same community (module), whereas **participation coefficient** likewise measures its connectivity to members of other communities (modules) relative to its own module. The high value of a z-score indicates the high within-cluster node degree. The participation coefficient of a node is close to 1 if the links from a node are equally distributed among all clusters and is equal to 0 if all links of a node are within its own cluster.

The node classification scheme applied in this work was defined previously (Guimera and Nunes Amaral, 2005) and can be summarised as follows. Based on the region in a parameter space of z-score and participation coefficient, nodes are categorised as hubs (with a higher number of links within its own community, $z \geq 2.5$) and non-hubs ($z < 2.5$). Non-hubs nodes are further assigned four different roles:

R1 - ultra-peripheral node (with all links within its community (module), $P \approx 0$),

R2 – peripheral node (if node has at least 60% its links within-community, 0.05 < P <0.625),

R3 - non-hub connector node (has half of its links, or at least 2 links, whichever is larger, within-community, 0.625 <P < 0.8)

R4 – non-hub kinless node (if a node has 35% of its links within-community, P > 0.8). Such nodes cannot clearly be assigned to one community. Thus, the node has links homogeneously spread among all communities.

The hub nodes, however, are divided into further three categories:

R5 – provincial hub (hub node with the great majority, at least 80%, of links within its community, P < 0.3),

R6 – connector hub (hub with many links to other clusters and at least half of its links within-community, 0.3 < P < 0.75)

R7 – global kinless hub (hub with links homogeneously spread among all clusters and fewer than half its links within-community, P > 0.75). As per R4 this identified that such nodes cannot clearly be assigned to one community.

Chi-square test of association

H_0 hypothesis - there is no association between the node position in the network and its effect on the pathogenic lifestyle

1. Initial contingency table:

a) Observed frequencies

**Node type**	**R1**	**R2**	**R3**	**R4**	**R5**	**R6**	**Row totals**
pathogenicity-related	109	258	144	25	1	2	539
pathogenicity-unrelated	351	214	117	8	10	0	700
**Colum totals**	460	472	261	33	11	2	1239

b) Expected frequencies

**Node type**	**R1**	**R2**	**R3**	**R4**	**R5**	**R6**
pathogenicity-related	200.11	205.33	113.54	14.36	4.79	0.87
pathogenicity-unrelated	259.89	266.67	147.46	18.64	6.21	1.13

Expected value is lower than 1 for R6 type node. Thus, we combined R5 and R6 categories

2. **Final contingency table used for the analysis:**

a) Observed frequencies

**Node type**	**R1**	**R2**	**R3**	**R4**	**R5 + R6**	**Row totals**
pathogenicity-related	109	258	144	25	3	539
pathogenicity-unrelated	351	214	117	8	10	700
**Colum totals**	460	472	261	33	13	1239

b) Expected frequencies

**Node type**	**R1**	**R2**	**R3**	**R4**	**R5 + R6**
pathogenicity-related	200.11	205.33	113.54	14.36	5.66
pathogenicity-unrelated	259.89	266.67	147.46	18.64	7.34

3. **Frequency table**

**Node type**	**LIFE-STYLE**	**O**	**E**	**O-E**	**$(O-E)^2$**	**(O-E)2/E**
R1	pathogenicity-related	109	200.11	-91.11	8301.58	41.48
R2	pathogenicity-related	258	205.33	52.67	2773.78	13.51
R3	pathogenicity-related	144	113.54	30.46	927.67	8.17
R4	pathogenicity-related	25	14.36	10.64	113.30	7.89
R5+R6	pathogenicity-related	3	5.66	-2.66	7.05	1.25
R1	pathogenicity-unrelated	351	259.89	91.11	8301.58	31.94
R2	pathogenicity-unrelated	214	266.67	-52.67	2773.78	10.40
R3	pathogenicity-unrelated	117	147.46	-30.46	927.67	6.29
R4	pathogenicity-unrelated	8	18.64	-10.64	113.30	6.08
R5+R6	pathogenicity-unrelated	10	7.34	2.66	7.05	0.96

O – observed frequencies, E – expected frequencies

4. **Chi-square test**

Rejected H_0 hypothesis that there is no association between the node position in the network and its effect on the pathogenic lifestyle

**χ^2**	127.97
**Critical value**	9.49
**d.f.**	4
**p-value**	1.0556E-26
**α**	0.05

5. Discrepancy table

Node type	(O-E)2/E	
	pathogenicity-related	pathogenicity-unrelated
R1: Ultra-peripheral node	(-) 41.48	(+) 31.94
R2: Peripheral node	(+) 13.51	(-)10.40
R3: non-hub-connector	(+) 8.17	(-) 6.29
R4: Non-hub kinless node	(+) 7.89	(-) 6.08
R5+R6: Provincial hub + connector-hub	(-) 1.25	(+) 0.96

Positive association between R2, R3, and R3 and pathogenicity-related nodes. Also, highly positive association between node type R1 and pathogenicity-unrelated nodes.

FORTUNATO, S. 2010. Community detection in graphs. *Physics Reports,* 486**,** 75-174.

FORTUNATO, S. & CASTELLANO, C. 2012. Community structure in graphs. *In:* MEYERS, R. A. (ed.) *Computational Complexity.* Springer New York.
