# Supplementary material for: PHI-Nets: A Network Resource for Ascomycete Fungal Pathogens to Annotate and Identify Putative Virulence Interacting Proteins and siRNA Targets
Source: Front Microbiol. 2019 Dec 6;10:2721. doi: 10.3389/fmicb.2019.02721 (PMC6908471; doi:10.3389/fmicb.2019.02721)

# Supplementary information 3

# Additional graphical display of *F. graminearum* nearest neighbour subnetworks

## Node color key for genes:

Magenta - mixed outcome where pathogen virulence is affected in some interactions but not others, pink (pathogenicity related), grey (pathogenicity unrelated), white (unknown phenotype).
Orange - *F. graminearum* orthologues of Bc siRNA target. Pathogen phenotypes taken from PHI-base version 4.6. Available gene identifiers and names are shown.

## Subnetwork A: see main Figure 5.

## Subnetwork 2:

## Subnetwork 3: Hsp90 overlapping subnetworks for 3 targets


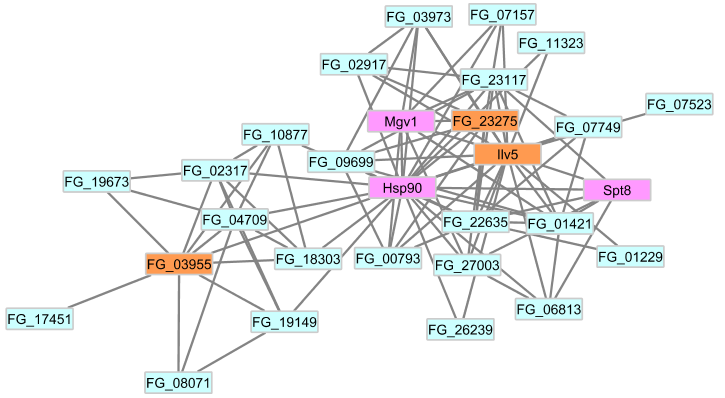


## Subnetwork 4: see Main Figure 6

## Subnetwork 5: FG_01625


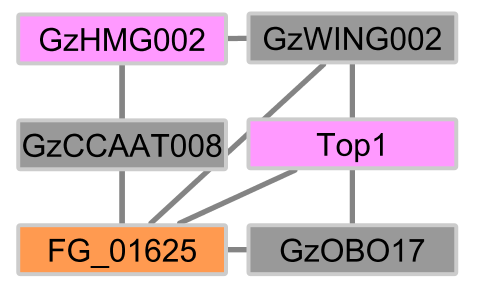


## Subnetwork 6: FG_23899


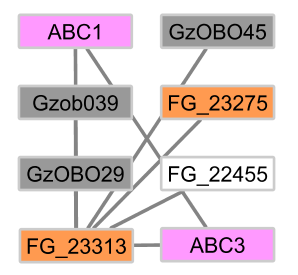

Supplement: INFORMATION S3 — Additional graphical display of B. cinerea and F. graminearum nearest neighbor subnetworks. [file Data_Sheet_3.docx]
